# RecQ mediated genome instability 2 (*RMI2*): a potential prognostic and immunological biomarker for pan-cancers

**DOI:** 10.18632/aging.204076

**Published:** 2022-05-12

**Authors:** Wei Wei, Xiaomei Ying, Liang Chen, Qingmei Sun, Xiaohuan Lu, Yang Xia, Rubin Xu, Zhechen Zhu, Dong Zhang, Qikai Tang, Li Li, Jiaheng Xie, Hongzhu Yu

**Affiliations:** 1 Department of General Surgery, Fuyang Hospital of Anhui Medical University, Fuyang 236000, Anhui, China; 2 Department of General Surgery, Suzhou Hospital of Anhui Medical University, Suzhou 234000, China; 3 Pancreas Center, The First Affiliated Hospital with Nanjing Medical University, Nanjing 210029, Jiangsu, China; 4 Department of Gastrointestinal Surgery, Union Hospital, Tongji Medical College, Huazhong University of Science and Technology, Wuhan 430022, Hubei, China; 5 Department of Immunology, School of Basic Medical Sciences, Anhui Medical University, Hefei 230032, Anhui, China; 6 Department of Burn and Plastic Surgery, The First Affiliated Hospital of Nanjing Medical University, Nanjing 210029, Jiangsu, China; 7 The State Key Lab of Reproductive, Department of Urology, The First Affiliated Hospital of Nanjing Medical University, Nanjing 210029, Jiangsu, China; 8 Department of Neurosurgery, The First Affiliated Hospital of Nanjing Medical University, Nanjing 210029, Jiangsu, China

**Keywords:** RMI2, pan-cancer, prognosis, immune infiltration, biomarker

## Abstract

Background: RecQ mediated genome instability 2 (*RMI2*) is an essential component of the BLM-TopoIIIa-RMI1-*RMI2* (BTR) complex. However, the mysterious veil of the potential immunological relationship of *RMI2* in tumorigenesis and development has not been revealed.

Methods: We conducted the differential expression (DE) analysis of the *RMI2* in pan-cancer using data onto Oncomine, TIMER, and GEPIA databases. Afterward, survival analysis and clinical-stage correlation analysis were performed via the TCGA database. Subsequently, we used R software to further explore the relationship between the expression level of *RMI2* and tumor mutation burden (TMB), microsatellite instability (MSI), tumor microenvironment (TME), tumor immune-infiltrated cells (TILs), immune checkpoints (ICP), mismatch repairs (MMRs) -related genes, m6A-related genes, DNA methylation-related genes. Finally, Gene Ontology (GO) and Kyoto Encyclopedia of Genes and Genomes (KEGG) functional networks were also performed for annotation via gene set enrichment analysis (GSEA).

Results: The *RMI2* expressed remarkably high in most cancer types compared to cancer adjacent normal tissues (*P* < 0.05). High expression of *RMI2* was linked to unfavorable prognosis and advanced stage of disease, especially in LIHC and PAAD. *RMI2* expression was related to TMB in 16 cancer types and MSI in 8 cancer types. Furthermore, it is significant positive correlations between *RMI2* and stromal and immune cells, ICP-related genes, MMRs-related genes, m6A-related genes, and DNA methylation-related genes. Finally, GSEA analysis revealed that *RMI2* was engaged in a variety of signaling pathways in pan-cancers.

Conclusions: *RMI2* may serve as a potential biological target and probably assume a crucial part in tumorigenesis and progression.

## INTRODUCTION

The BTR complex, of which *RMI2* is a part, maintains genome stability and has a significant impact on DNA replication and its damage repairs [1]. *RMI2* works to synergizes with RMI1 and topoisomerase III alpha to maintain replication fork stability. At the same time, it can also dissolve double Holliday junctions to prevent genome instability. It has been reported that the downshift of *RMI2* can cause mild Bloom syndrome disease features [2].

In recent years, numerous studies have emerged to reveal the relationship between *RMI2* and cancers. *RMI2* can affect cell function by up-regulating runt-related transcription factor 2(RUNX2). In parallel, it can also increase downstream of RUNX2 downstream molecule SLUG by regulating EMT, thereby regulating the invasion and migration of lung cancer [3]. It was reported that *RMI2* served a positive impact on the growth of hepatocellular carcinoma (HCC) and inhibits its apoptosis by regulating the p53 signaling pathway [4]. Overexpression of *RMI2* will result in a significant down-regulation of p53, p21, PUMA, and Gadd45. Liu et al. found that the high expression of *RMI2* affects the tumorigenesis of cervical cancer in a variety of ways [5]. In a nutshell, abnormal expression of *RMI2* has implications on DNA replication, repair, and cell metabolism. These studies suggest that the expression level of *RMI2* is closely associated with tumor appreciation, invasion, and migration.

In recent years, TME and immune infiltration are the focus of tumor research, which is extremely important for understanding the oncogenesis and developing corresponding immunotherapy. However, the relationship between *RMI2* and immunology in cancer is still vague. Further, exploring the immune-related relationship of *RMI2* and tumors is urgently required. In this research, comprehensive, analyzed the survival relationship of *RMI2* expression in multiple cancer types. And also we explored the correlation of *RMI2* expression with TMB, MSI, tumor microenvironment, tumor- and immune-related genes, methyltransferases, and MMRs-related genes. Finally, we explored the possible functions and pathways of *RMI2* via GSEA.

## RESULTS

### The expression level of *RMI2* in pan-cancer

**Figure 1:**
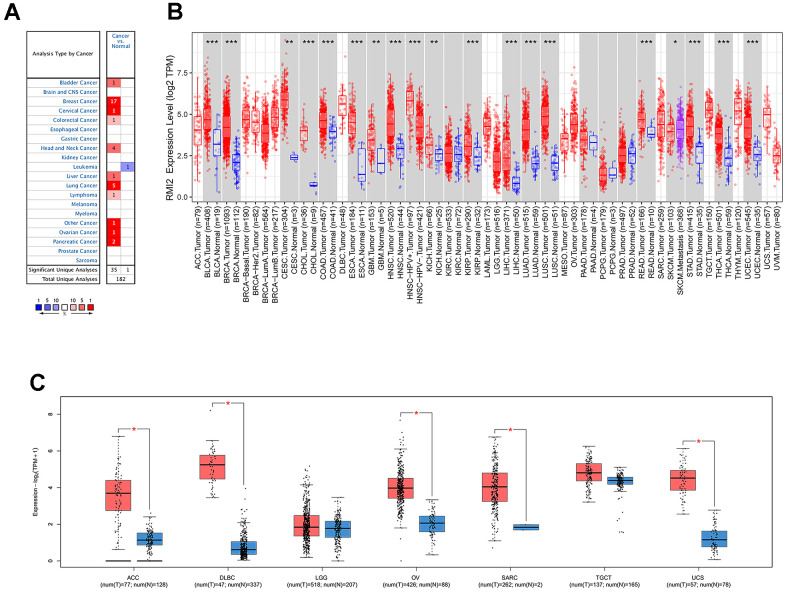
**The differential expression of *RMI2* gene in human tumors.** (**A**) The expression of *RMI2* in different cancers and paired normal tissue in the Oncomine database. (**B**) The *RMI2* expression levels in different cancer types from the TCGA database analyzed by the TIMER database. ((″***″ indicates P<0.001, ″**″ indicates P<0.01 and ″*″ indicates P<0.05). (**C**) The *RMI2* expression in several cancers and adjacent paired normal tissue in the GEPIA database.

Oncomine database results show that *RMI2* mRNA is significantly differentially expressed in normal and cancerous tissues in pan-cancerous. As shown in Figure 1A, high expression of *RMI2* was correlated with 10 cancers (bladder, breast, cervical, colorectal, head and neck, liver, lung, lymphoma, ovarian, pancreatic) and significantly lower expression only in leukemia. Next, the TIMER was utilized to validate the expression of the *RMI2* gene in 33 cancers. Not surprisingly, *RMI2* was significantly highly expressed in 17 cancer types (BLCA, BRCA, CHOL, COAD, ESCA, HNSC, KICH, KIRC, KIRP, LIHC, LUAD, LUSC, PRAD, READ, STAD, THCA, and UCEC) compared to normal tissues and was only low expressed in one cancer type, SKCM (Figure 1B and Supplementary Table 2). The p-value for each comparison is indicated by an asterisk (“***” indicates *P*<0.001, “**” indicates *P*<0.01 and “*” indicates *P*<0.05). Since some tumor types of the above figure did not have corresponding normal samples, we further used the GEPIA database to match TCGA tumor and GTEx normal data, in order to ensure the comprehensiveness of the study. The results of GEPIA analysis indicate that *RMI2* was also highly expressed in five tumors (ACC, DLBC, OV, SARC, and UCS) (Figure 1C). In combination, these results revealed that *RMI2* is aberrantly overexpressed up to 22 cancer types. In combination, we found that *RMI2* was significantly overexpressed in 22 tumor types.

### Correlation of *RMI2* expression and OS

**Figure 2:**
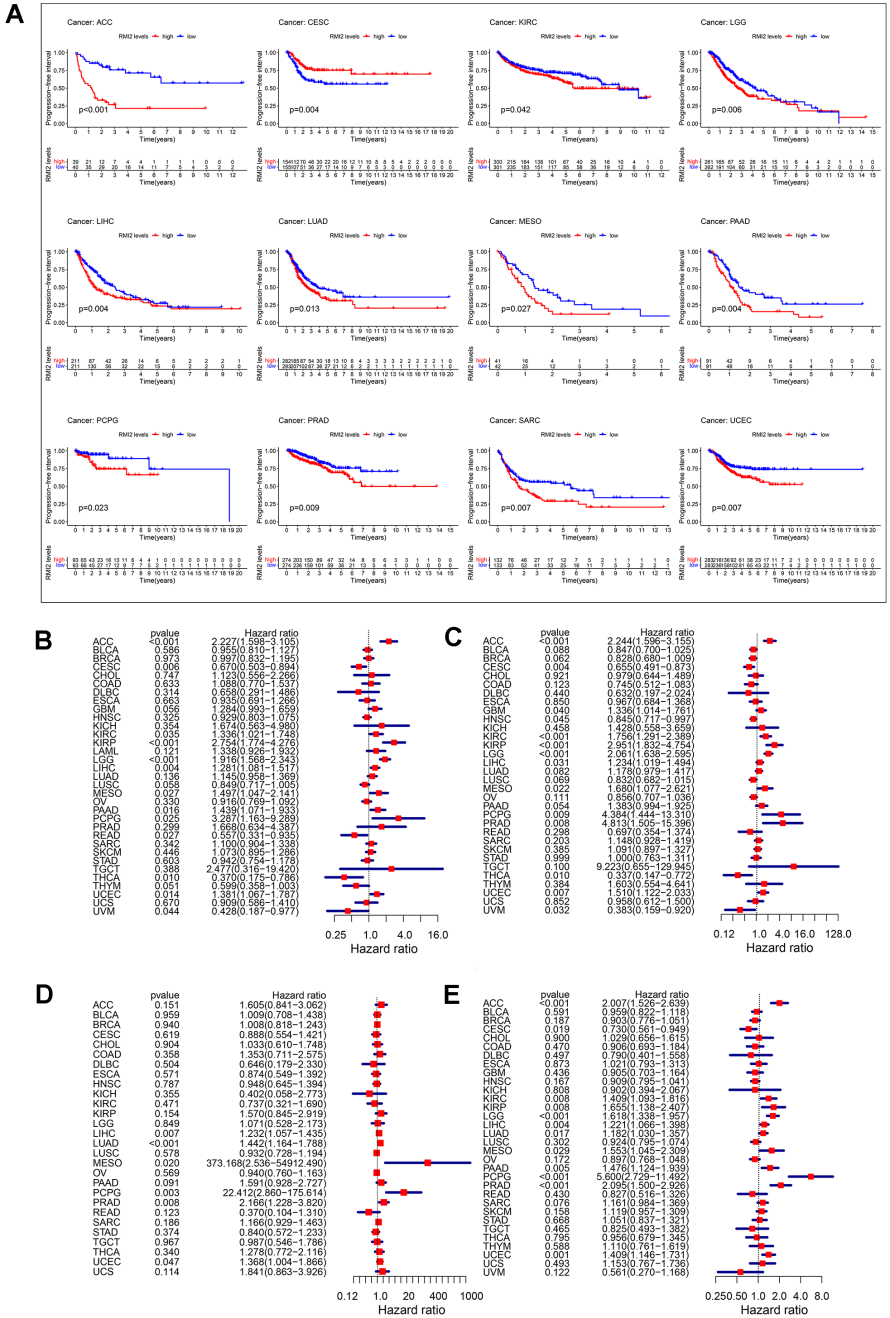
Correlation between *RMI2* expression in patients with DFI (**A**). (**A**) Survival analyses of *RMI2* expression via the Kaplan–Meier PFI curves in ACC, CESC, KIRC, LGG, LIHC, LUAD, MESO, PAAD, PCPG, PRAD, SARC and UCEC. Cox proportional risk model was used to study the effect of *RMI2* on the prognosis of multiple human tumors (**B**–**E**). (**B**) Effect of *RMI2* on OS in 33 human tumors. (**C**) Effect of *RMI2* on DSS in 33 human tumors. (**D**) Effect of *RMI2* on DFS in 33 human tumors. (**E**) Effect of *RMI2* on PFI in 33 human tumors.

We have analyzed the relationship between *RMI2* expression and OS by analyzing gene expression transcripts and clinical data. KM survival analysis results revealed that high *RMI2* expression was related to low OS time in 7 cancers, including ACC, GBM, KIRP, LGG, LIHC, MESO, and PAAD. And only in CESC, *RMI2* low expression was associated with low OS time (Supplementary Figure 1A). Meanwhile, the COX analysis also revealed that *RMI2* expression was associated with OS in 13 cancer types. Meanwhile, the univariate COX regression also showed that *RMI2* expression was associated with OS in 13 cancers. Among them, it was associated with a high risk of survival in 9 cancers (ACC, KIRC, KIRP, LGG, LIHC, MESO, PAAD, PCPG, and UCEC), and low risk of survival in 4 cancers (CESC, READ, THCA, UVM) (Figure 2B).

### Correlation of *RMI2* expression and DSS

To reflect the deaths identified as tumor factors during follow-up, we analyzed the relationship between *RMI2* expression and DSS in TCGA 33 cancers. KM analysis revealed that high expression of *RMI2* was associated with low OS time in 7 cancers, including ACC, GBM, KIRC, LGG, LIHC, MESO, PAAD. Only in CESC and UVM, the high expression of *RMI2* was related to the prolongation of DSS time (Supplementary Figure 1B). At the same time, the results of DSS analysis by the univariate COX regression revealed that the expression of *RMI2* was a risk factor in ACC, GBM, KIRC, KIRP, LGG, LIHC, MESO, PCPG, PRAD, UCEC. *RMI2* expression was only a protective factor in four cancer types (CESC, HNSC, THCA, and UVM) increasing the DSS of patients (Figure 2C).

### Correlation of *RMI2* expression and DFI

Then, we also evaluated the relationship between *RMI2* and DFI for 33 cancers in the TCGA database. The results of KM analysis revealed that the high expression of RMI was not related to the high DFI of tumors (Supplementary Figure 1C), but related to the low DFI of 3 kinds of tumors (LIHC, LUAD, PAAD). The COX analysis revealed that among the five tumor types (LIHC, LUAD, MESO, PCPG, PRAD) were associated with a high risk of survival (Figure 2D).

### Correlation of *RMI2* expression and PFI

In addition, we explored the relationship between *RMI2* and PFI in 33 kinds of tumors in the TCGA database. Kaplan-Meier curve results showed that the high expression of *RMI2* was related to the time of low PFI in 11 kinds of cancers, including ACC, KIRP, LGG, LIHC, LUAD, MESO, PAAD, PCPG, PRAD, SARC, UCEC. Coincidentally, it is consistent with the results of OS, only in CESC, the low expression of *RMI2* is related with low survival (Figure 2A). The COX analysis showed that among the 11 cancers (ACC, KIRC, KIRP, LGG, LIHC, LUAD, MESO, PAAD, PCPG, PRAD, UCEC), it was associated with a high risk of tumors. Consistent with the above results, *RMI2* expression was only associated with low tumor risk in CESC cancer (Figure 2E).

### Correlation of *RMI2* expression and clinicopathologic characteristics

**Figure 3:**
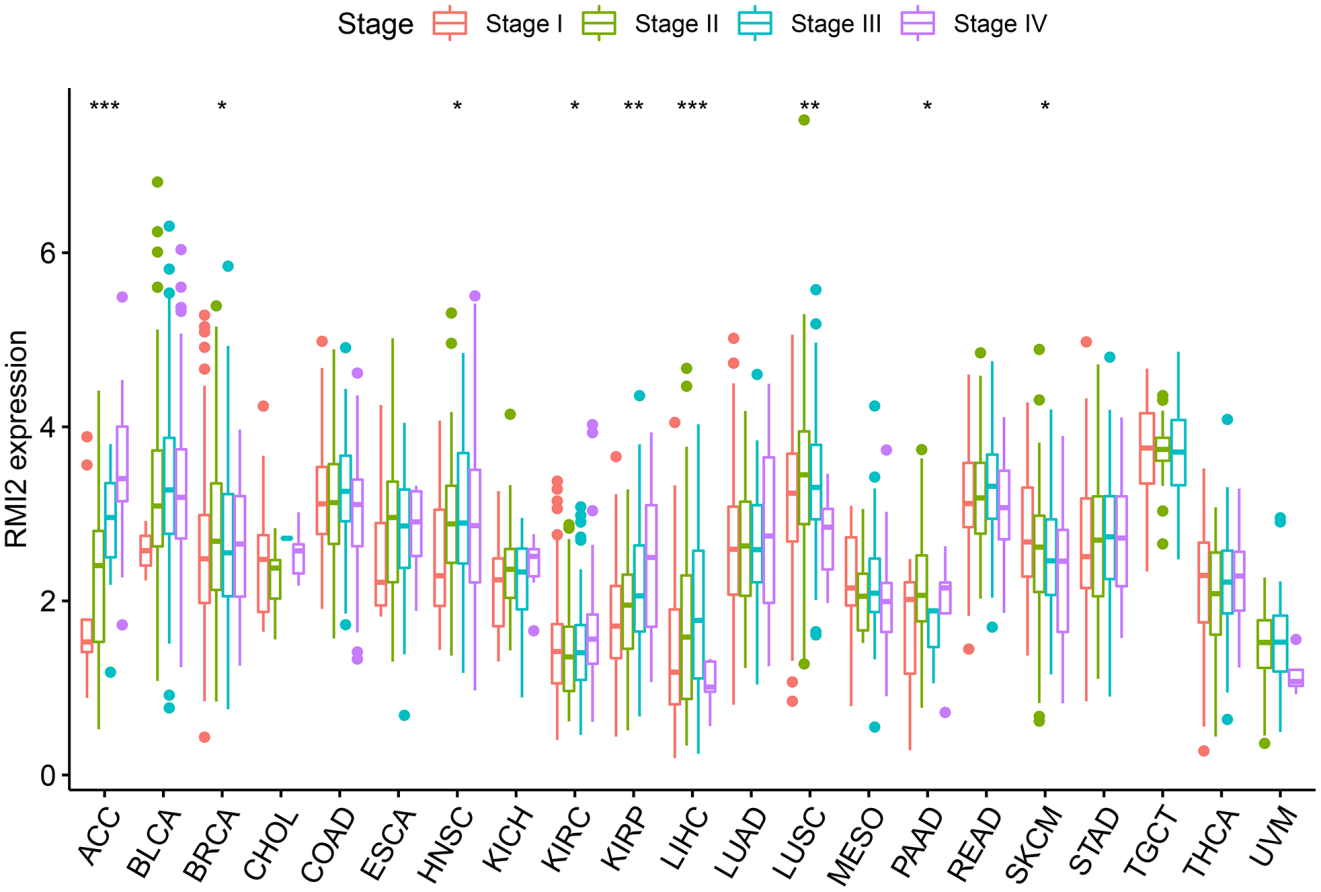
The correlations between *RMI2* expression and pathological stages in various cancers.

Overall, Usually, patients with late clinical-stage of tumor have poorer survival prognosis We analyzed the relevance between the expression of *RMI2* and clinicopathological stage. Figure 3 revealed that the expression of *RMI2* differed significantly with tumor stage in the nine tumor types (ACC, BRCA, HNSC, KIRC, KIRP, LIHC, LUSC, PAAD, SKCM) (*P*<0.05). The expression of *RMI2* increased with rising tumor grades in most tumor types.

### Correlation of *RMI2* expression and TMB, MSI

**Figure 4:**
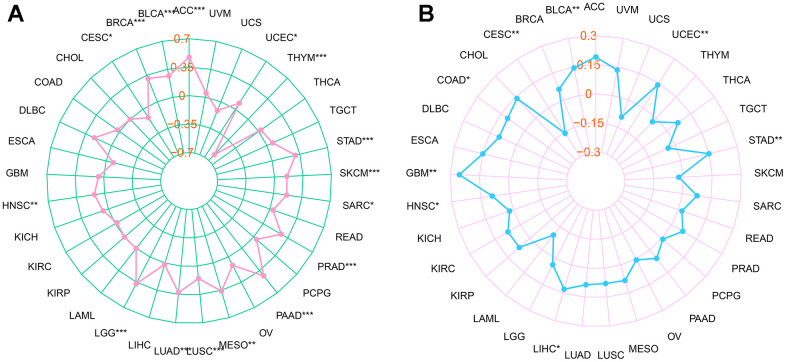
**Relationship between *RMI2* expression, TMB and MSI in pan-cancer.** (**A**) The relationship between TMB and *RMI2*. (**B**) The relationship between MSI and *RMI2*. Spearman rank correlation test, p <0.05 was regarded as the statistical criteria to set thresholds.

The total number of substitutions and insertion/deletion mutations per trillion bases in the exon coding region of the evaluated gene in the tumor sample can be used as a concept for TMB. Research on TMB and tumor prognosis and immunotherapy are becoming more and more in-depth, and more correlations are being uncovered [6]. Therefore, we calculated the TMB of each type of cancer to study the relationship between *RMI2* expression and TMB. The detailed information is shown in Supplementary Table 3. We found that the expression of *RMI2* was significantly correlated with 16 cancer types, of which 14 cancer types (ACC, UCEC, STAD, SKCM, SARC, PRAD, PAAD, MESO, LUSC, LUAD, LGG, HNSC, BRCA, BLCA) were positively correlated, but negatively correlated with THYM and CESC (Figure 4A). MSI refers to the phenomenon of microsatellite sequence length change caused by insertion or deletion mutation during DNA replication caused by mismatch repair (MMR) function defects, and MSI may be related to the occurrence of cancer. We found that in the seven cancer types (BLCA, GBM, STAD, UCEC, COAD, HNSC, LIHC) the expression of *RMI2* was positively correlated with MSI-related genes. There is only a negative correlation in CESC (Figure 4B and Supplementary Table 4).

### Correlation of *RMI2* expression and TME, TILs and ICP genes

After the immune score and stromal score were calculated for each tumor sample, we were astonished by the intertwined association between *RMI2* expression and immune/stromal score.

**Figure 5:**
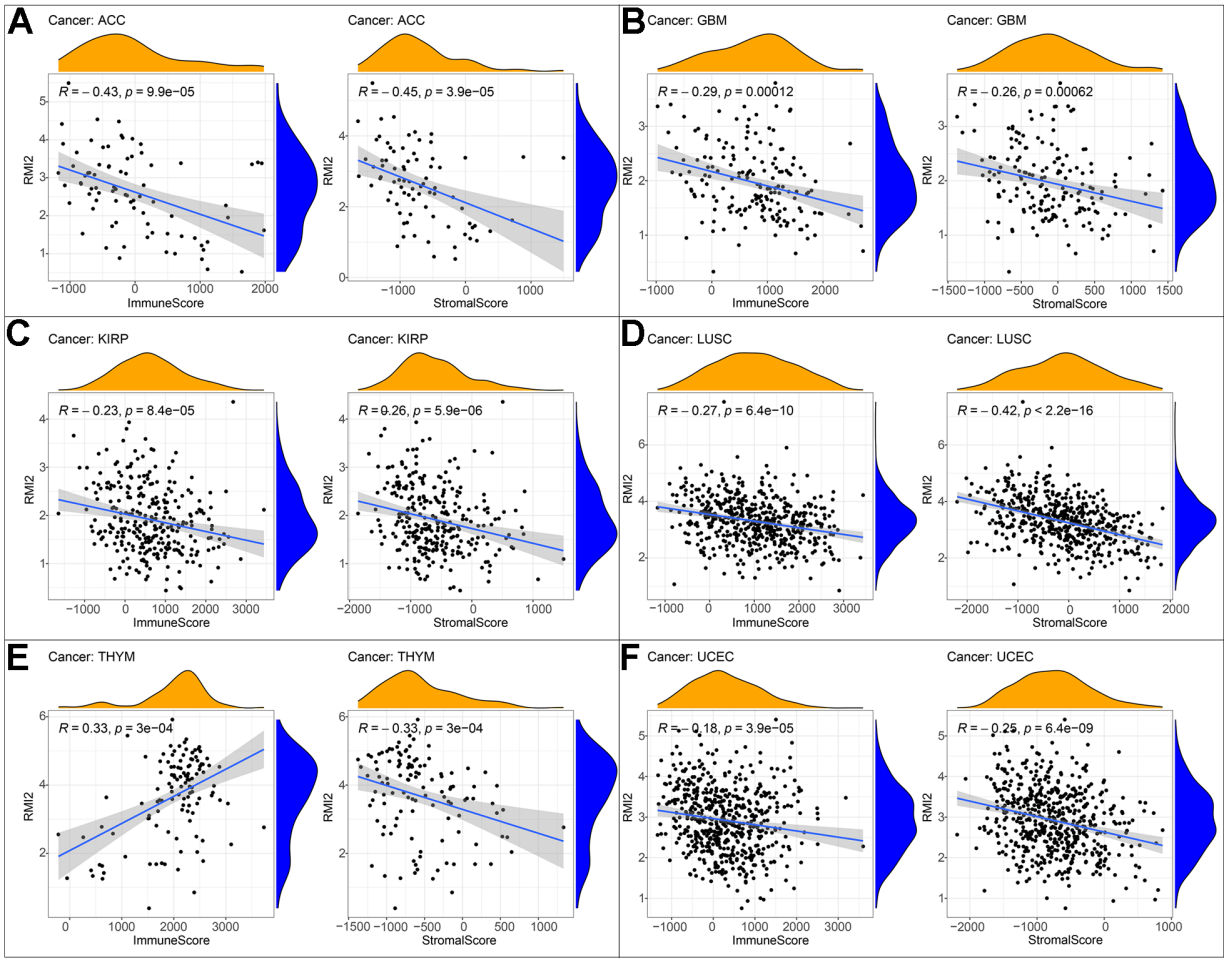
**Correlation of *RMI2* expression with ImmuneScore and StromalScore in various cancers.** (**A**) Correlation of *RMI2* expression with ImmuneScore and StromalScore in ACC. (**B**) Correlation of *RMI2* expression with ImmuneScore and StromalScore in GBM. (**C**) Correlation of *RMI2* expression with ImmuneScore and StromalScore in KIRP. (**D**) Correlation of *RMI2* expression with ImmuneScore and StromalScore in LUSC. (**E**) Correlation of *RMI2* expression with ImmuneScore and StromalScore in THYM. (**F**) Correlation of *RMI2* expression with ImmuneScore and StromalScore in UCEC.

**Figure 6:**
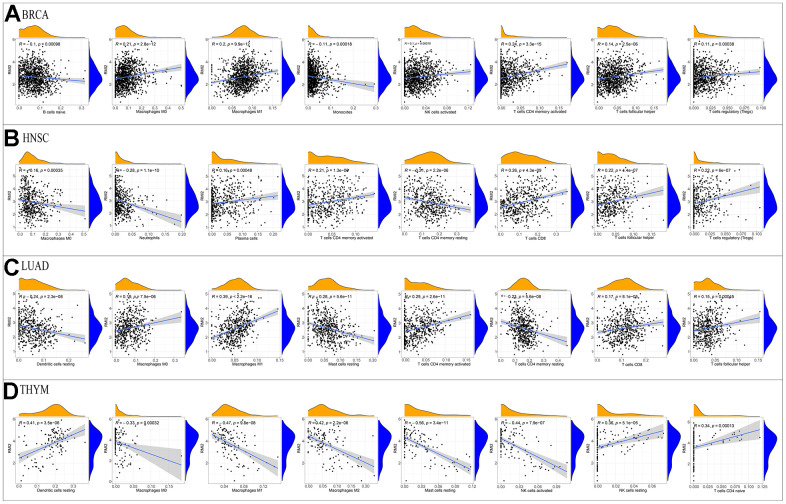
**Correlation of RMI2 expression with immune infiltration level in the four tumor types.** Correlation Between RMI2 expression and immune infiltration level in (**A**) BRCA, (**B**)HNSC, (**C**) LUAD, (**D**)THYM.

**Figure 7:**
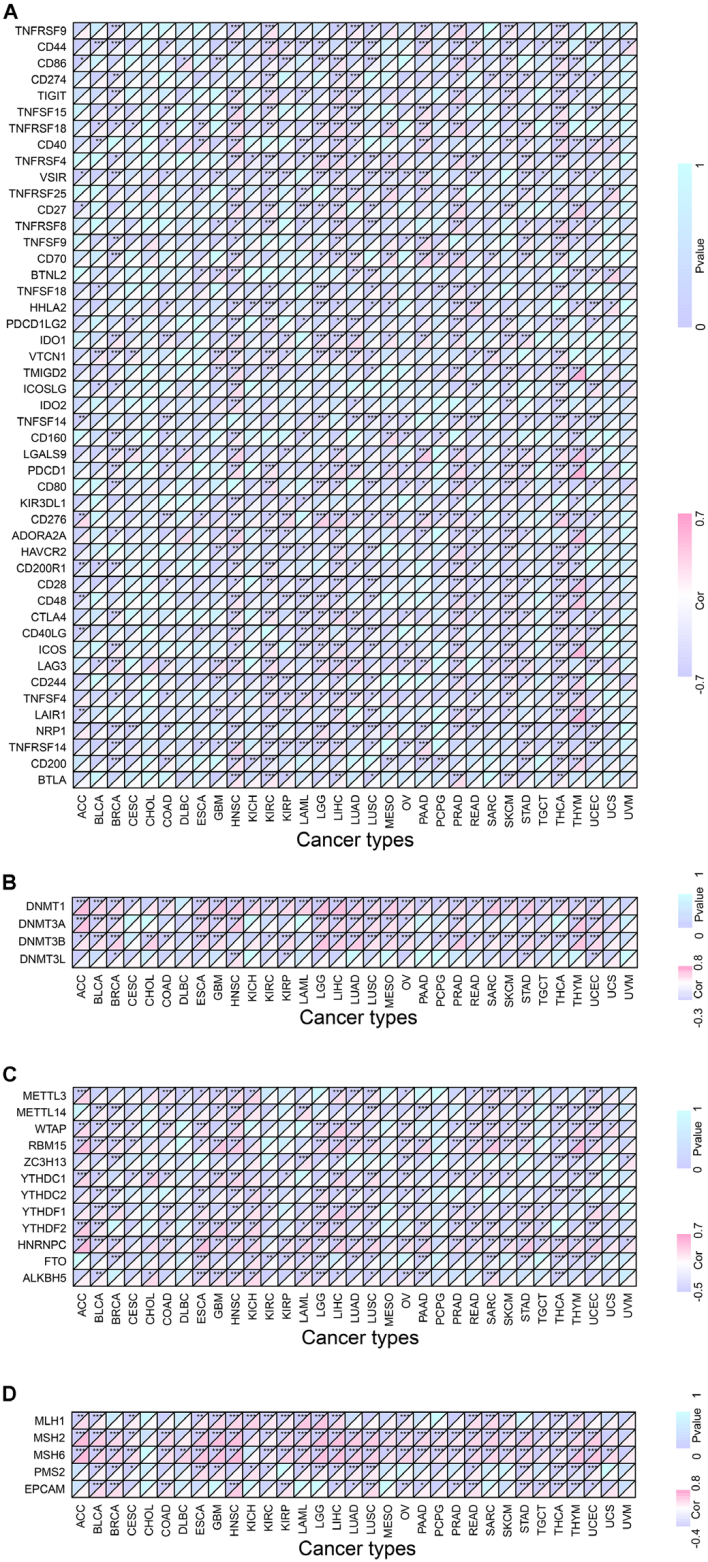
**Co-expression of *RMI2* with certain specific genes in 33 tumor types.** (**A**) Co-expression of *RMI2* with ICP related genes. (**B**) Co-expression of *RMI2* with DNA methyltransferases. (**C**) Co-expression of *RMI2* with m6A related genes. (**D**) Co-expression of *RMI2* with MMRs genes.

In addition to a positive correlation with the immune score of THYM and a negative correlation with its stromal score, the expression level of *RMI2* was negatively correlated with the immune score and stromal score of ACC, GBM, KIRP, LUSC, and UCEC (Figure 5A–5F, Supplementary Figure 2 and Supplementary Table 5). One of the independent predictors of tumor-primary lymph node status and survival is tumor-infiltrating lymphocytes. Analysis of TILs confirmed that *RMI2* expression was intertwined and entangled with the level of immune infiltration in different tumor types. Figure 6 showed that the strong correlation between *RMI2* and BRCA, HNSC, LUAD, THYM. The detailed results of 33 cancer types are shown in the Supplementary Figures 3, 4 and Supplementary Table 6. The relationship between more than 40 ICP-related genes and *RMI2* expression in a variety of cancers was developed in this study. The results are striking and *RMI2* is associated with gene expression of multiple ICPs in many types of cancer (Figure 7A and Supplementary Table 7). For example, it is related to 39 ICP genes in HNSC, 32 ICP genes in KIRC, 35 ICP genes in LIHC, 36 ICP genes in PRAD and 38 ICP genes in THCA. In short, the critical role of *RMI2* in the immune infiltration and immune escape of multiple tumors cannot be ignored.

### Co-expression of *RMI2* with DNA methyltransferases, m6A and MMRs

Recent studies have found that DNA methyltransferases are intertwined with tumorigenesis [7], and DNA methylation detection technique is helpful for early screening of tumors [8]. We found that the expression of *RMI2* was inextricably linked to DNMT1 and DNMT3A, DNMT3B and DNMT3 methyltransferases genes in a variety of tumors (Figure 7B and Supplementary Table 8). Among them, BRCA, STAD, UCEC are associated with these methyltransferase-related genes. *RMI2* expression and M6A-related genes were studied after demonstrating that more than 8 cancer types (BLCA, ESCA, HNSC, LIHC, LUSC, OV, SARC and UCEC) with a strong correlation with m6A related genes (Figure 7C and Supplementary Table 9). We found that MLH1, MSH2, MSH6, PMS2, and EPCAM MMRs genes are closely related to *RMI2* genes. Figure 7D showed that BLCA, LIHC, READ, THCA, and THYM were closely related to 5 MMRs genes. Among them, 19 cancer types are associated with 4 or more MMRs genes. The detailed information is in the Supplementary Table 10.

### GSEA analysis

**Figure 8:**
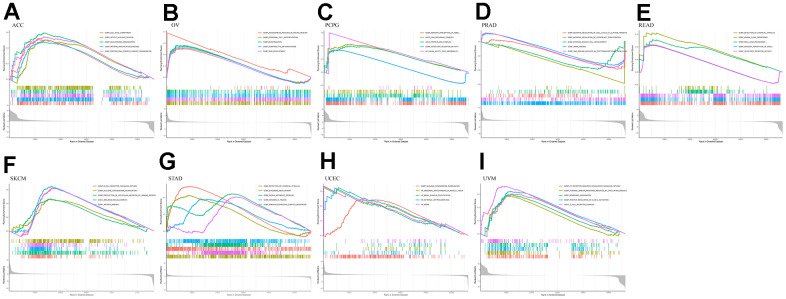
**GO enrichment plots from GSEA in various cancers.** P<0.05 and enrichment results of more than 5 were considered for shown. (**A**) Enrichment results of GO function in cancer ACC. (**B**) Enrichment results of GO function in cancer OV. (**C**) Enrichment results of GO function in cancer PCPG. (**D**) Enrichment results of GO function in cancer PRAD. (**E**) Enrichment results of GO function in cancer READ. (**F**) Enrichment results of GO function in cancer SKCM. (**G**) Enrichment results of GO function in cancer STAD. (**H**) Enrichment results of GO function in cancer UCEC. (**I**) Enrichment results of GO function in cancer UVM.

Using GO analysis of the high and low *RMI2* gene expression groups, we found that the ACC group with high *RMI2* expression was mainly associated with cell division. In the high expression group of the *RMI2* gene in OV, it was mainly related to epithelial cell division. In PCPG, *RMI2* is mainly concerned with DNA synthesis. In PRAD, the main enrichment functions in the *RMI2* low expression group were related to the cell cycle. In READ, the primary enrichment function is related to the senses. In SKCM, the main enrichment function in the *RMI2* high expression group was related to immunity. In STAD, the enriched functions in the high expression group of the *RMI2* gene were mainly related to metabolism. In UCEC, the high expression of the *RMI2* gene may be associated with mitochondrial abnormalities. In UVM, it may be associated with immune cell communication in the *RMI2* high expression group (Figure 8, Supplementary Figure 5 and Supplementary Table 11).

**Figure 9:**
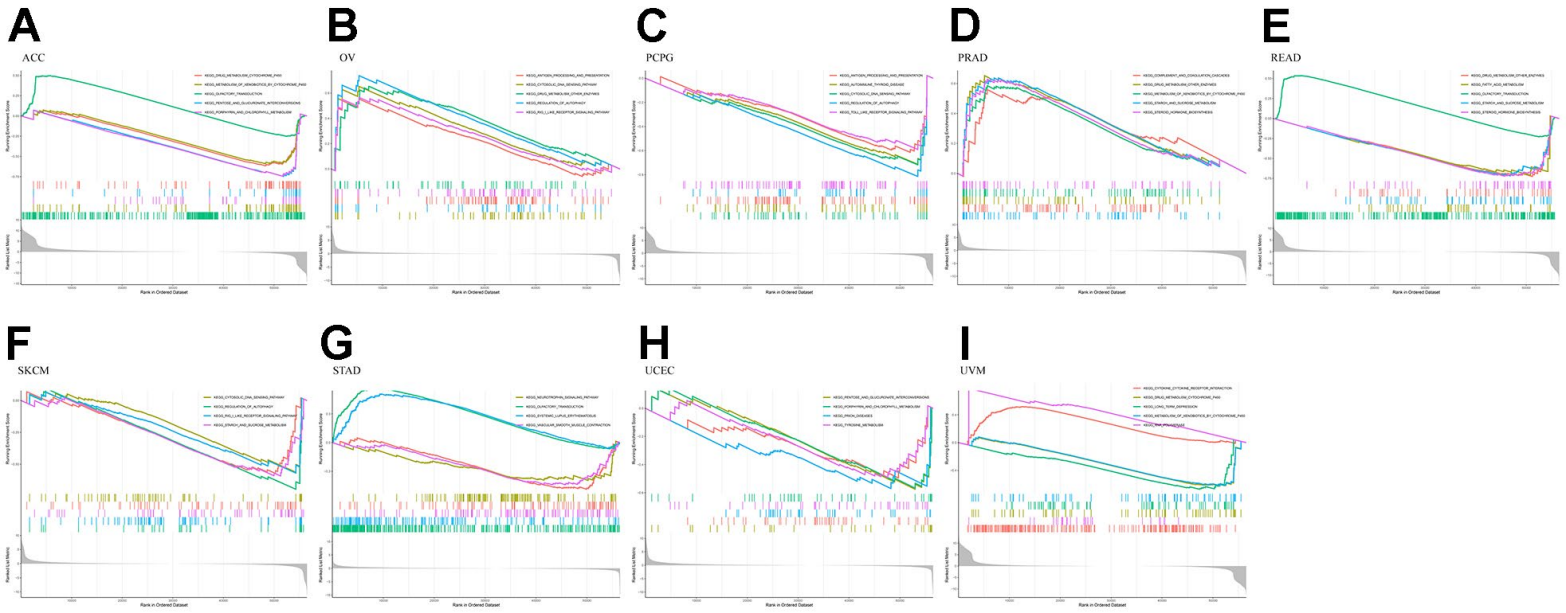
**KEGG enrichment plots from GSEA in various cancers.** P<0.05 and enrichment results of more than 5 were considered for shown. (**A**) Enrichment results of KEGG pathway in cancer ACC. (**B**) Enrichment results of KEGG pathway in cancer OV. (**C**) Enrichment results of KEGG pathway in cancer PCPG. (**D**) Enrichment results of KEGG pathway in cancer PRAD. (**E**) Enrichment results of KEGG pathway in cancer READ. (**F**) Enrichment results of KEGG pathway in cancer SKCM. (**G**) Enrichment results of KEGG pathway in cancer STAD. (**H**) Enrichment results of KEGG pathway in cancer UCEC. (**I**) Enrichment results of KEGG pathway in cancer UVM.

At the same time, through further analysis of KEGG, it was found that in the PCPG gene low-expression group, the main enriched pathways were related to immunity, such as antigen presentation and autoimmune pathways. In PRAD, the pathways enriched in the group with high expression of the *RMI2* gene were mainly related to metabolism. In READ, the main enriched pathway in the *RMI2* low expression group was related to metabolism. ACC, OV, SKCM, UCEC, and UVM were mainly enriched in the metabolic pathway in the *RMI2* low expression group. In STAD, the pathways enriched in the high *RMI2* expression group may be related to autoimmune diseases, while the pathways enriched in the low *RMI2* expression group are mainly related to neurotrophic and olfactory pathways (Figure 9 and Supplementary Table 12).

### The validation of *RMI2* gene in hepatocellular carcinoma was performed in ICGC database

**Figure 10:**
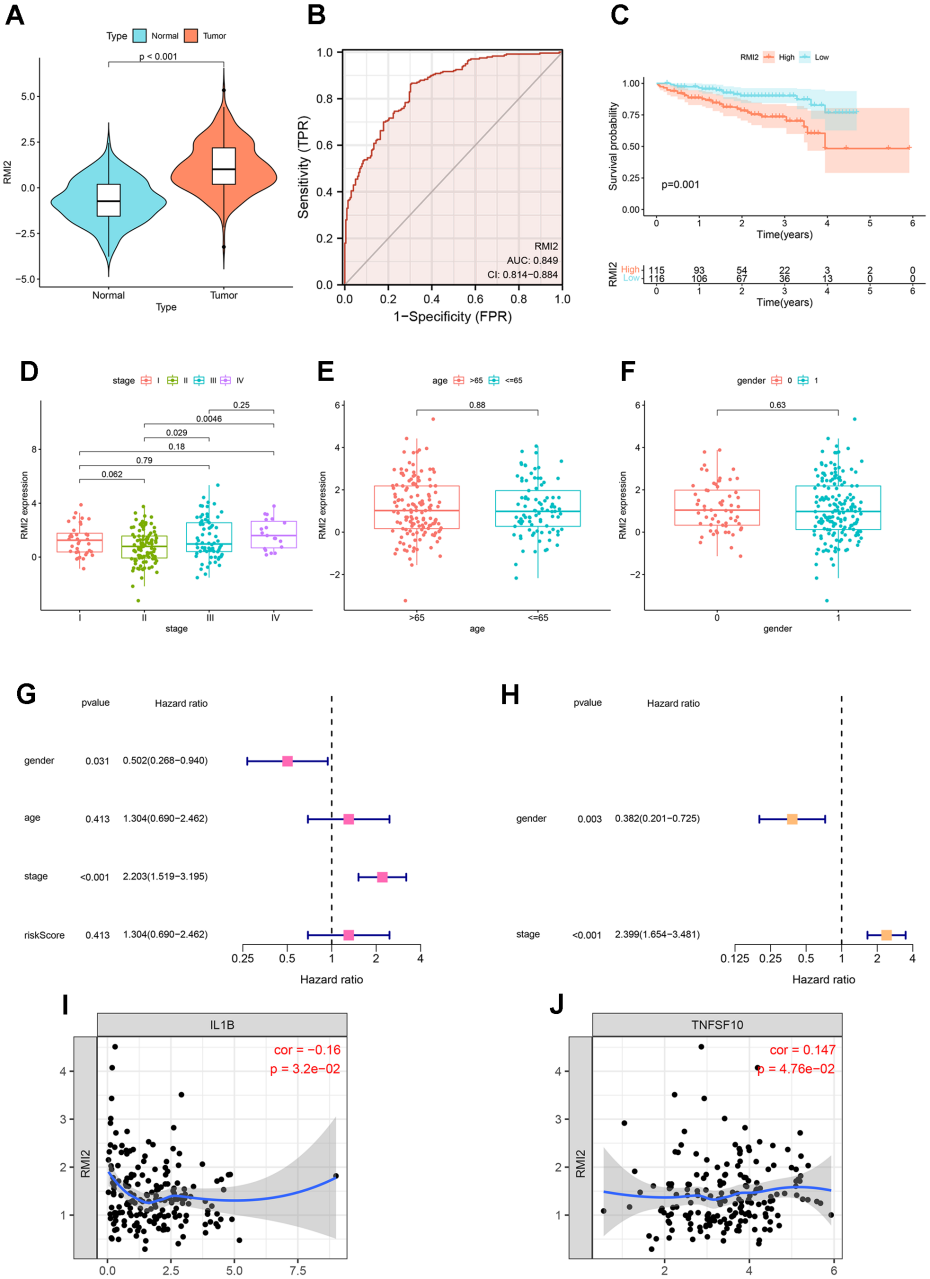
**Further validation of *RMI2* in other databases.** (**A**) Expression analysis in ICGC database showed that *RMI2* was up-regulated in hepatocellular carcinoma (P<0.001). (**B**) ROC curve showed that the AUC value of *RMI2* in hepatocellular carcinoma was 0.849. (**C**) Survival analysis showed that high *RMI2* expression was associated with poor prognosis of hepatocellular carcinoma. (**D**–**F**) The correlation between *RMI2* and stage, age, and gender of HCC patients. (**G**) Univariate COX regression. (**H**) Multivariate COX regression. (**I**, **J**) *RMI2* was significantly correlated with IL1B and TNFSF10 (P<0.05, Figure 10I, 10J).

Hepatocellular carcinoma samples from the ICGC database were used to validate the results. There were 202 normal liver tissue samples and 240 hepatocellular carcinoma samples. First, expression analysis showed that *RMI2* was up-regulated in hepatocellular carcinoma (*P*<0.001, Figure 10A). ROC curve showed that the AUC value of *RMI2* in hepatocellular carcinoma was 0.849 (Figure 10B). Survival analysis showed that high *RMI2* expression was associated with poor prognosis of hepatocellular carcinoma (*P*=0.001, Figure 10C). Subsequently, the correlation between *RMI2* and stage, age, and gender of HCC patients was shown (Figure 10D–10F). Univariate COX regression was used to explore independent prognostic indicators of HCC (Figure 10G). Multivariate COX regression was used for further verification (Figure 10H). The TIMER database was used to explore the correlation between *RMI2* and immunomodulators in PCPG. The results showed that *RMI2* was significantly correlated with IL1B and TNFSF10 (*P*<0.05, Figure 10I, 10J).

## DISCUSSION

Globally, cancer is a critical disease responsible for death which killing nearly 10 million people in 2020. Patients who suffer from cancer may bear both psychological [9] and physical [10] pressures. At the same time, it is these malignant diseases that impose a heavy financial burden on families and healthcare systems [11]. However, the pathogenesis of cancer is extremely complex [12, 13] and it is difficult to monitor it in the early stage. Further, the traditional diagnosis and treatment methods are not ideal in early diagnostic methods and therapies. Fortunately, in recent years, the study of immune-related targets may bring a glimmer of hope for the early monitoring and treatment of tumors [14]. Immune-related research on pan-cancer-related targets may find a new direction [15] for early diagnosis or personalized treatment of cancer. Further, it may make a minor contribution to reducing the physical and mental suffering of patients and the burden of cancer on the national public health system. The function of the BTR complex formed by *RMI2* is complex. It can participate in the control of DNA crossover-formation, gene replication, and repair after damage, which is crucial in maintaining the stability of the genome [1]. In recent research, *RMI2* has attracted wide attention in the field of cancer research, such as liver cancer [4], lung cancer [3], cervical cancer [5], and prostate cancer [16]. The overexpression of *RMI2* is significantly related to the metastatic progression of some tumors or unfavorable prognosis. It may serve as a potential pre-detection target or therapeutic biomarker. However, the research on *RMI2* only stays in a limited number of cancer types, and the relationship between its role in pan-cancer and immunology is still vague.

In the current research, we comprehensively demonstrated, the expression level of *RMI2* and its immunological relationship to multiple cancer types. We found that there was an abnormally high expression of *RMI2* in 22 cancer types. *RMI2* expression level is correlated with the unfavorable prognosis of patients with various types of tumors (OS, DSS, PFI, and DFI), and the late clinical stage is related to the high expression level. Meanwhile, the expression of *RMI2* has closely associated with TMB, MSI, MMR, and DNA methylation. Furthermore, this study demonstrates that the expression of *RMI2* in many tumors (especially in ACC, GBM, KIRP, LUSC, UCEC) was negatively correlated with the immune score and stromal score. The expression level of *RMI2* is related to a variety of immune infiltrating cells. To sum up, *RMI2* may become a biomarker and provide some new ideas about tumor immunotherapy of cancer.

According to previous studies [17, 18], DNA methylation plays an important role in many diseases. For example, immune diseases and cancer. DNA methylation of the promoter GpC island is involved in the carcinogenesis and development of stomach cancer [19]. It regulates gene expression (DNA methylation and its basic function) by regulating proteins that are suppressed by genes or inhibiting transcription factors that bind to DNA. Zhou et al. showed that gene methylation plays a crucial role in the diagnosis of head and neck cancers. Methylation of some genes is an independent prognostic marker of HNSCC [17]. Our study found that *RMI2* is related to DNA methylation in multiple types of cancer, among which BRCA, STAD, UCEC is associated with four DNA methylation genes (DNMT3L, DNMT3B, DNMT3A, DNMT1). N6-methyladenosine (m6A) is widely involved in the internal modification of RNA in eukaryotic cells and plays a crucial role in RNA metabolism and a variety of biological processes. In acute myeloid leukemia (AML), METTL14 regulates MYB and MYC via M6A modification, and plays a carcinogenic role in regulating cell self-renewal and inhibiting bone marrow differentiation [20]. Studies by Xiao Li et al. have shown that knockout of METTL3 gene in renal cell carcinoma can promote cell proliferation, migration, and invasion via PI3K-Akt-mTOR or EMT pathways, and induce G0 / G1 phase arrest to regulate cell cycle [21]. However, few were aware that *RMI2* expression and the genes related to m6A. Our study found that the *RMI2* gene is associated with multiple M6A genes in BLCA, ESCA, HNSC, LIHC, LUSC, OV, SARC, UCEC, which is helpful for the development of drugs aimed at M6A related genes. However, further experiments are needed to study the relationship between *RMI2* expression and M6A. Suppressing ICPs related to immune escape is one of the methods of immunotherapy [22]. In recent years, there have been more and more studies on tumor immunity, and the results have been remarkable [23, 24]. Some studies have achieved clinical transformation. For example, multiple countries have approved programmed death 1 (PD-1) or programmed death-ligand 1 (PD-L1) inhibitors for the treatment of lung, melanoma, and breast cancer. A study has demonstrated that ICI may increase the sensitivity of recurrent/metastatic squamous cell carcinoma of the head and neck (R/M SCCHN) to chemotherapy [25]. In addition, this study has shown that the expression of *RMI2* is related to a variety of ICP genes. Especially in cancer types such as HNSC, KIRC, LIHC, PRAD, and THCA.

The cellular components, immune cells, and stromal cells of TME can affect the growth and differentiation of tumor cells [26]. Numerous studies have indicated that multiple types of tumor-infiltrating lymphocytes [27, 28], such as tumor-infiltrating neutrophils (TINS) [29] and tumor-associated macrophages (TAMS) [30], can affect the growth of tumor cells and then affect the prognosis of patients with a variety of mechanisms. Non-invasively predict the tumor-infiltrating immune cells of high-grade gliomas (HGG) through radiomic signatures, and use the absolute quantitative level as a prognostic indicator of HGG [31]. Previous studies have shown that tumor-infiltrating immune cells (TIICs) and T cell activation are closely related to the survival of breast cancer patients [32]. Therefore, regulating the level of TILs provides a new direction for tumor immunotherapy. A variety of immune cells may promote or inhibit tumor progression through different mechanisms. Our research also found that the expression of *RMI2* was mainly negatively related to the scores of the immune score and stromal score of TME, such as ACC, GBM, KIRP, LUSC, UCEC, and other cancer types. This shows that the higher the expression of *RMI2*, the fewer immune cells and stromal cells in TME. *RMI2* may inhibit or promote the progression of cancer by aggregating and regulating immune infiltrating cells. In breast cancer [33], gastric cancer, colorectal cancer [34], high macrophage infiltration is associated with poor prognosis. NKs can use death receptors to induce apoptosis and perforin/granzyme to induce cytotoxicity and then kill tumor cells. The increased risk of cancer is related to the decrease of NK cell activity. [35]. CD4 Treg cells harm anti-tumor immunity by inhibiting tumor-associated antigens. Different immune infiltrating cells are complex and changeable in the occurrence and development of tumors, so for different tumors, the study of the relationship between *RMI2* and immune infiltration-related cells can promote tumor immunity and drug development and therapy. Our current study found that *RMI2* expression was significantly associated with TILs in BRCA, HNSC, LUAD, THYM, such as B cells, CD4+ T cells, CD8+ T cells, T cells follicular helper, T cells regulatory (Tregs), NK cells, Macrophages, Dendritic cells, Mast cells, and other immune cells. Some drugs based on TME and immune infiltration have been approved for the treatment of tumors. Therefore, according to the immune infiltration of different tumors, the corresponding immunotherapy can be developed, and anti-tumor therapy is very important.

However, some limitations still exist in this study. This study found that the expression of *RMI2* was related to TME, immune infiltration, and ICPs of pan-cancer, but there are no *in vivo* or *in vitro* experiments directly proved that *RMI2* affects the survival of tumor patients through these immunological mechanisms. Secondly, we have carried out the integration and evaluation analysis of several databases to obtain the integrity of the results, but the gene chip and sequencing data of different databases may be different, and the way the data are collected may be biased. So it may cause some bias of the research results to some extent. Finally, the exact mechanism by which *RMI2* occurs to tumor-associated immunity is still unclear. In the future, study the tumor immune mechanism for specific tumor types in order to explore the relevant mechanism is necessary, and it is helpful to the research and development of anti-tumor drugs. Generally speaking, our study on the relationship between *RMI2* and pan-cancer prognosis and immunology provides a new idea for the immune-related treatment of cancer and may be able to do our part to alleviate the physical and mental pain of tumor patients and the national public health financial burden. In the future, more attention may be paid to the study of the immunological mechanism of *RMI2* in cancer, and the research on tumor immunity may benefit from this.

## CONCLUSIONS

Returning to the previous question posed in the Introduction, it is now possible to state that *RMI2* may predict the prognosis and treatment of cancer as a potential biomarker. The high expression lever of *RMI2* is related to many tumor types, and the poor prognosis and disease progression of tumors are related to its expression, especially in LIHC, PAAD. The abnormal expression of *RMI2* is negatively correlated with immune cells and stromal cells in most types of cancer, and it is closely related to B cells, CD4 T cells, CD8 T cells, T cells follicular helper and other TILs and a variety of immune-related genes (ICP, MMRs, m6A). And the enrichment of multiple pathways is also related to it.

## MATERIALS AND METHODS

### Data collection and processing

Oncomine is a database for oncology (http://www.oncomine.org/resource/login.html), which integrates RNA and DNA-seq data profiles from sources TCGA, GEO, and other data that has been made public. To analyze the differential expression of the *RMI2* gene in pan-cancer, fold change ≥1.5 and *P*-value ≤0.05 were set in the Oncomine database. RNA transcript data, mutation data, and clinical data for 33 tumors in the TCGA database were obtained from the University of California Santa Cruz (UCSC) Xena browser (https://xena.ucsc.edu/). The R software package (R version: 4.1.1) was used for data screening and processing. And the data onto the analysis about *RMI2* in the TCGA database for different patient samples were listed in Supplementary Table 1. The abbreviation and sample size of 33 cancer types of TCGA database were listed in Table 1. TIMER database (http://timer.comp-genomics.org/) mainly provides a presentation of the results of TCGA data analysis, and it can be used to validate the results of differential expression analysis. For cancer types that failed to be matched to normal samples of the TCGA database, we again used the GEPIA (http://gepia.cancer-pku.cn/) database Match TCGA tumor and GTEx normal to facilitate differential expression analysis.

**Table 1:** The abbreviation and sample size of 33 cancer types of TCGA database.

**TCGA cancer abbreviation**	**TCGA cancer type**	**Total sample number**
ACC	Adrenocortical carcinoma	79
BLCA	Bladder Urothelial Carcinoma	430
BRCA	Breast invasive carcinoma	1217
CESC	Cervical squamous cell carcinoma and endocervical adenocarcinoma	309
CHOL	Cholangiocarcinoma	45
COAD	Colon adenocarcinoma	512
DLBC	Large B-cell Lymphoma	48
ESCA	Esophageal carcinoma	173
GBM	Glioblastoma multiforme	173
HNSC	Head and Neck squamous cell carcinoma	546
KICH	Kidney Chromophobe	89
KIRC	Kidney renal clear cell carcinoma	607
KIRP	Kidney renal papillary cell carcinoma	321
LAML	Acute Myeloid Leukemia	151
LGG	Brain Lower Grade Glioma	529
LIHC	Liver hepatocellular carcinoma	424
LUAD	Lung adenocarcinoma	585
LUSC	Lung squamous cell carcinoma	550
MESO	Mesothelioma	86
OV	Ovarian serous cystadenocarcinoma	379
PAAD	Pancreatic adenocarcinoma	182
PCPG	Pheochromocytoma and Paraganglioma	186
PRAD	Prostate adenocarcinoma	551
READ	Rectum adenocarcinoma	177
SARC	Sarcoma	265
SKCM	Skin Cutaneous Melanoma	472
STAD	Stomach adenocarcinoma	407
TGCT	Testicular Germ Cell Tumors	156
THCA	Thymoma	568
THYM	Thyroid carcinoma	121
UCEC	Uterine Corpus Endometrial Carcinoma	583
UCS	Uterine Carcinosarcoma	56
UVM	Uveal Melanoma	80

### Kaplan-Meier (KM) survival and Cox regression analysis

KM Survival analysis was utilized in evaluating the survival rate of the two groups, which has divided the patients into the high-risk group and low-risk group according to the median expression value of *RMI2* (*p*<0.05). Cox regression was performed to compare *RMI2* gene expression with OS, DSS, DFI, and PFI. These items include Hazard ratio (HR), P-values from the log-rank, and 95% confidence intervals set via the maximum selected log-rank statistic, which is based on the grouping of *RMI2* gene expression levels. The “forestplot” and “survival” R packages were adopted for Cox analysis and plotting.

### TMB and MSI correlation analysis

We performed the processing of somatic mutation data from the TCGA by Perl (Perl version: 5.32.1) to count the TMB scores of 33 tumor types and each patient sample.

Spearman rank-sum test was performed to evaluate the relevance between *RMI2* expression and TMB and MSI. The “fmsb” R package was used to produce a visual analysis of the radar plot.

### Methyltransferase, m6A, and MMRs related genes analysis

The expression levels of DNMT1, DNMT3A, DNMT3B, and DNMT3 genes related to methylation have been obtained into the TCGA database. Moreover, previous studies have shown that mismatch repairs(MMRs) have a tight relationship with cancer development. The “limma” R package was used to evaluate the link between the expression levels of five MMRs genes (MLH1, MSH2, MSH6, PMS2, and EPCAM) and *RMI2*. The same data processing scheme was used to analyze whether there was a specific association between *RMI2* and these two items (DNA methyltransferase as well as m6A-related gene expression levels) in the TCGA. The Spearman correlation method was chosen to assess the relationship among *RMI2* and these three indicators (DNA methyltransferases, m6A and MMRs). The “ggplot2”, “ggpubr”, and “ggExtra” R packages were used for statistical analysis and to graph heat maps.

### Immune correlation analysis

In recent years, an expanding series of studies have found that tumor immunity is deeply involved in the occurrence and advancement of tumors. We analyzed the two tumor immunity projects, Immune Microenvironment and Immune Cell Infiltration, which are related to the *RMI2* gene. At the same time, its relationship with immune checkpoint-related genes was investigated. The “estimate” R package was chosen to determine the StromalScore and ImmuneScore for each of the 33 types of cancer in the TCGA. The “ e1071” R package was also used to analyze the relationship between various immune-related cell infiltrations in 33 cancers. Spearman’s correlation method was chosen to assess the correlation analysis of *RMI2* gene expression with two immune microenvironmental indicators (StromalScore, ImmuneScore). The method was also chosen to identify the correlation between various types of TILs and checkpoint genes.

### GO and KEGG pathway analysis

The three R packages “enrichplot”, “org.Hs.eg.db”, and “clusterProfiler” were used for GO and KEGG enrichment analysis and visualization.

### Statistical analysis

The Perl (5.32.1) script was used to screen the expression of *RMI2* and sample data in pan-cancer. R software (4.1.1) is used for statistical analysis and graphing. Kaplan-Meier method and Cox Regression method were utilized to analyze the relationship between the expression of *RMI2* and patient survival and risk factors. Spearman rank-sum test was utilized to assess the correlation between *RMI2* expression and TMB and MSI. Spearman correlation analysis was utilized to assess the relationship between *RMI2* expression and Methyltransferase, MMR, and immune checkpoint-related genes. We accept such result that R>0.20 is regarded as a positive correlation and *P*<0.05 is regarded as statistically significant.

### Data availability statement

Our data is collected from public databases and from the following websites:

http://genome-asia.ucsc.edu/index.html; http://www.oncomine.org/resource/login.html; http://timer.cistrome.org/; http://gepia2.cancer-pku.cn/#index.

### Ethics statement

Ethics approval and written consent were not needed for this study exploring the public data. The ethics committee of Fuyang Hospital affiliated to Anhui Medical University agreed to the ethical waiver.

## Supplementary Material

Supplementary Figures

Supplementary Table 1

Supplementary Tables 2-5

Supplementary Table 6

Supplementary Table 7

Supplementary Table 8

Supplementary Table 9

Supplementary Table 10

Supplementary Table 11

Supplementary Table 12
