# Supplementary material for: RecQ mediated genome instability 2 (RMI2): a potential prognostic and immunological biomarker for pan-cancers
Source: Aging (Albany NY). 2022 May 12;14(9):4107–36. doi: 10.18632/aging.204076 (PMC9134953; doi:10.18632/aging.204076)
Supplement: Supplementary Figures [file aging-14-204076-s001.pdf]

SUPPLEMENTARY FIGURES

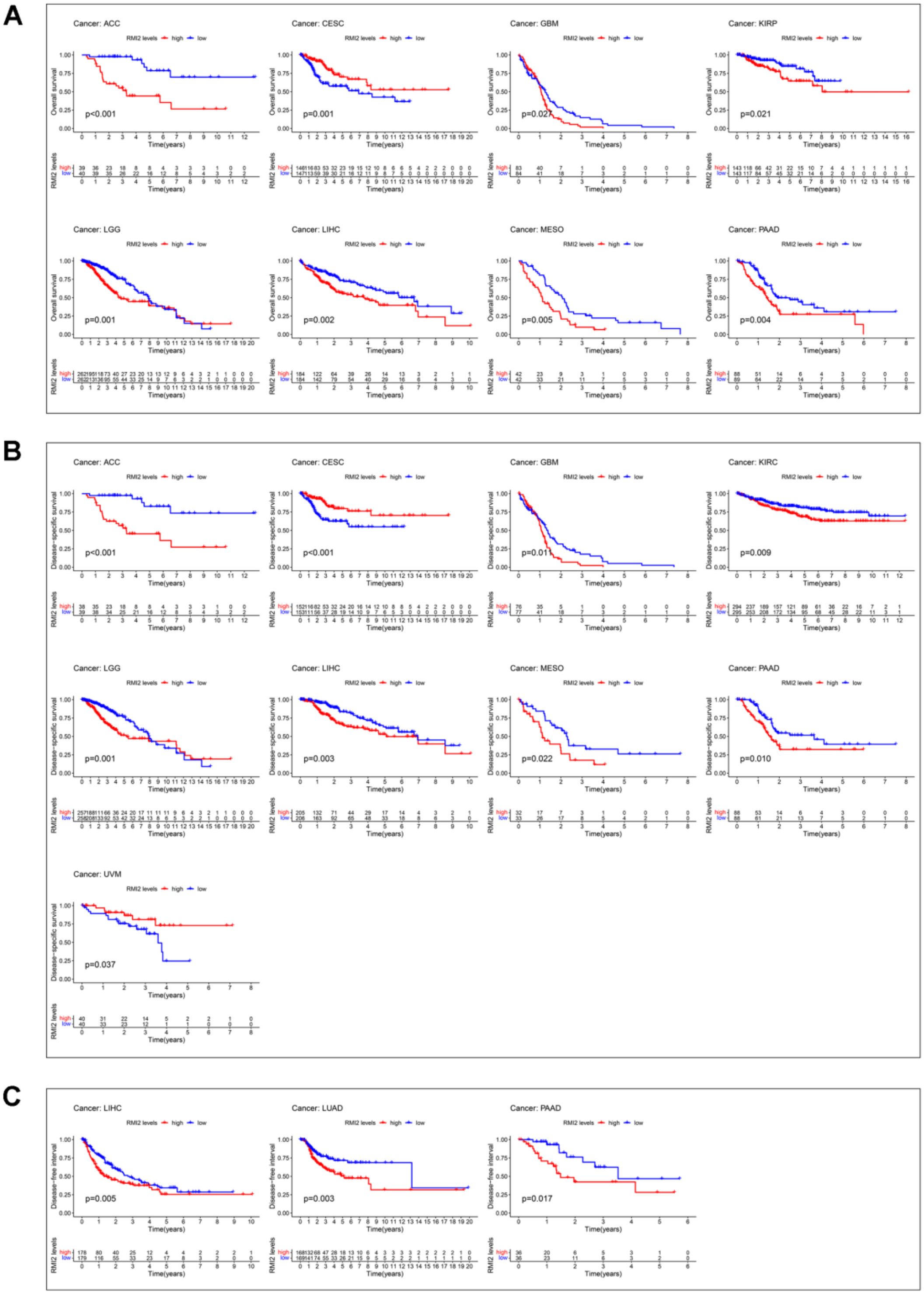

Supplementary Figure 1. Correlation between *RMI2* expression in patients with OS, PFI, DSS, and DFI. (**A**–**C**) (**A**) Survival analyses of *RMI2* expression via the Kaplan–Meier OS curves in ACC, CESC, GBM, KIRP, LGG, LIHC, MESO and PAAD. (**B**) Survival analyses of *RMI2* expression via the Kaplan–Meier DSS curves in ACC, CESC, GBM, KIRC, LGG, LIHC, MESO, PAAD, UVM. (**C**) Survival analyses of *RMI2* expression via the Kaplan–Meier DFI curves in LIHC, LUAD and PAAD.

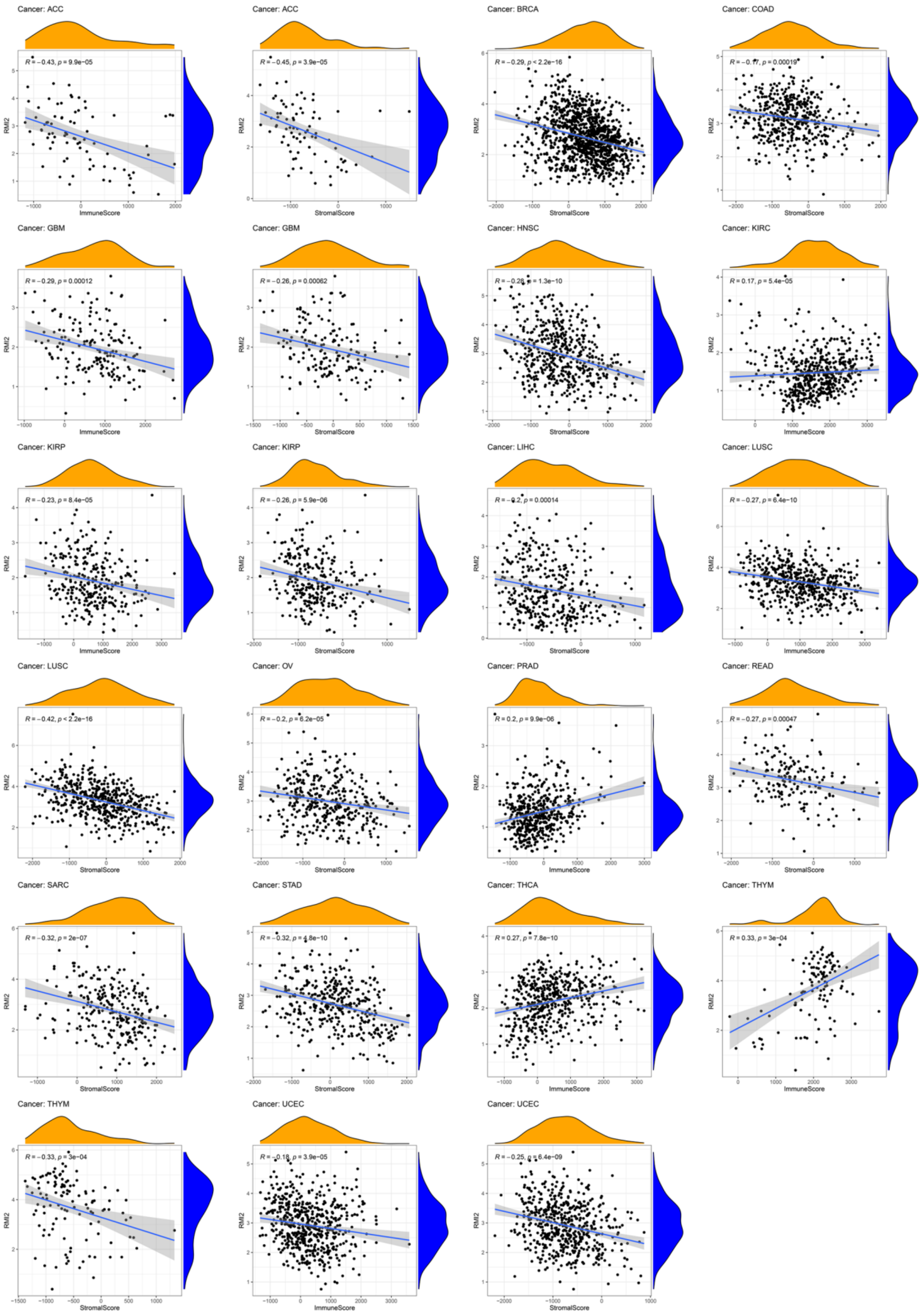

Supplementary Figure 2. Correlation between *RMI2* and ImmuneScore and StromalScore in pan-cancer(P<0.05).

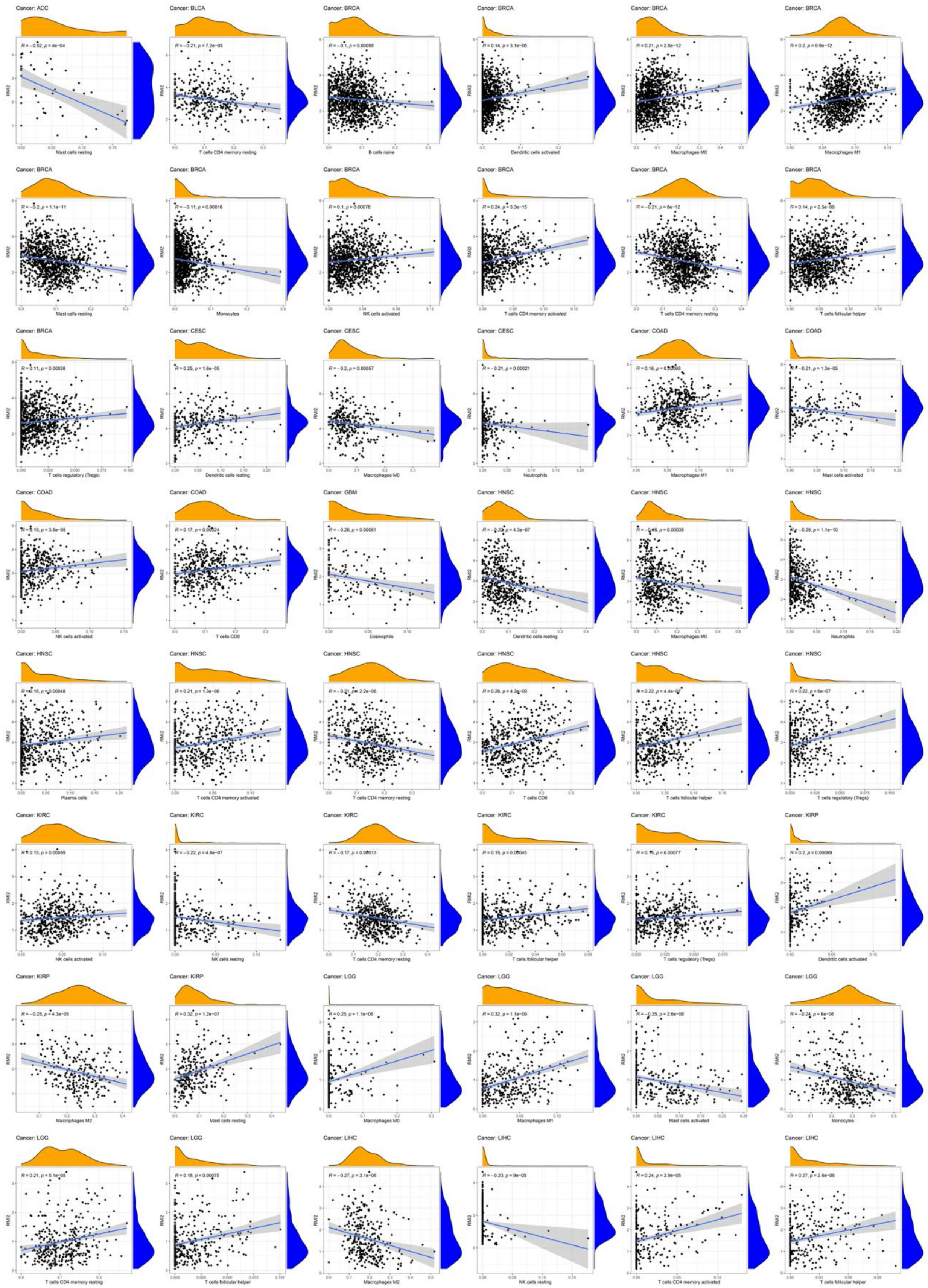

Supplementary Figure 3. The relationship between *RMI2* and specific immune infiltrating cells in pan-cancer(P<0.05).

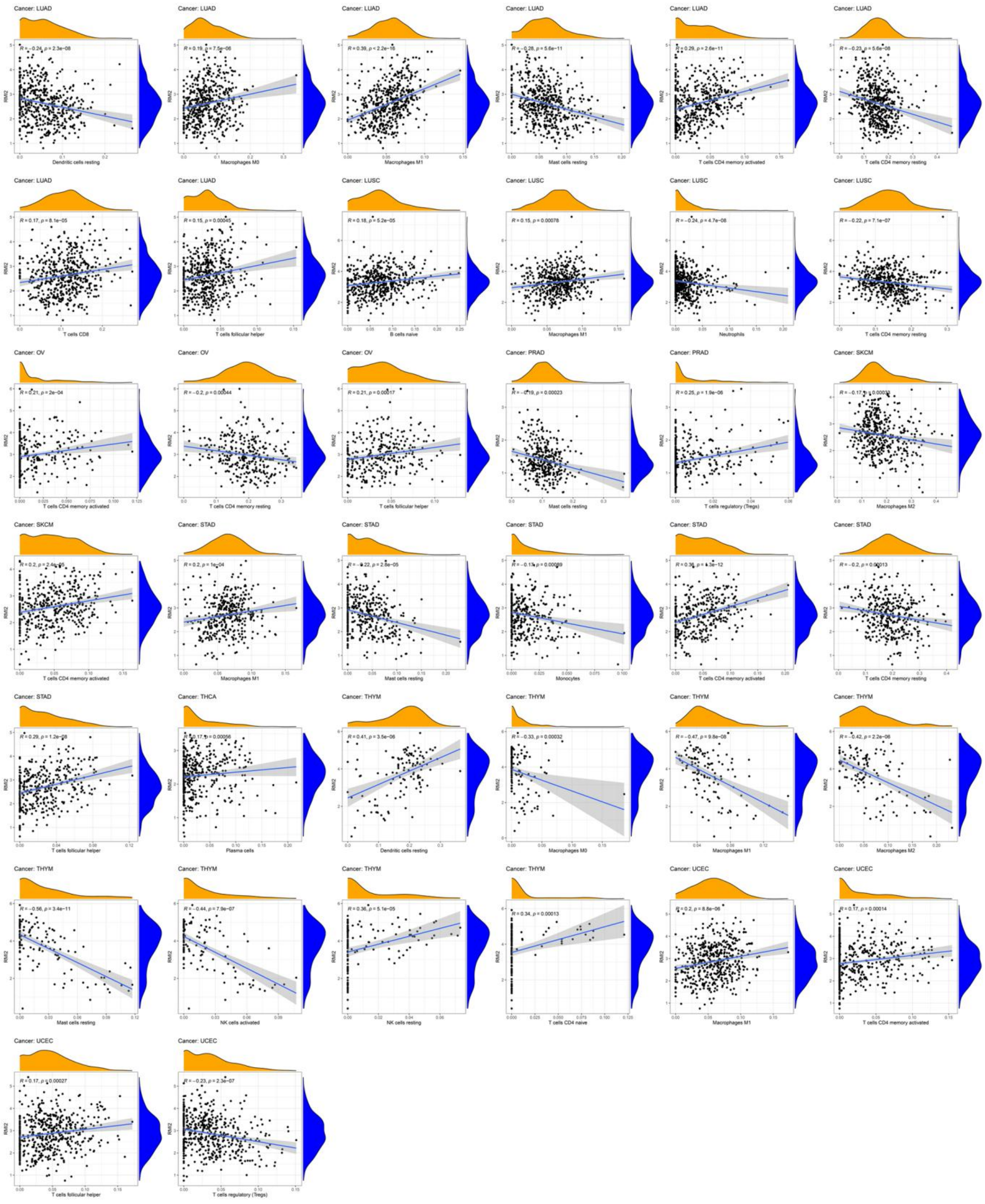

Supplementary Figure 4. The relationship between *RMI2* and specific immune infiltrating cells in pan-cancer.

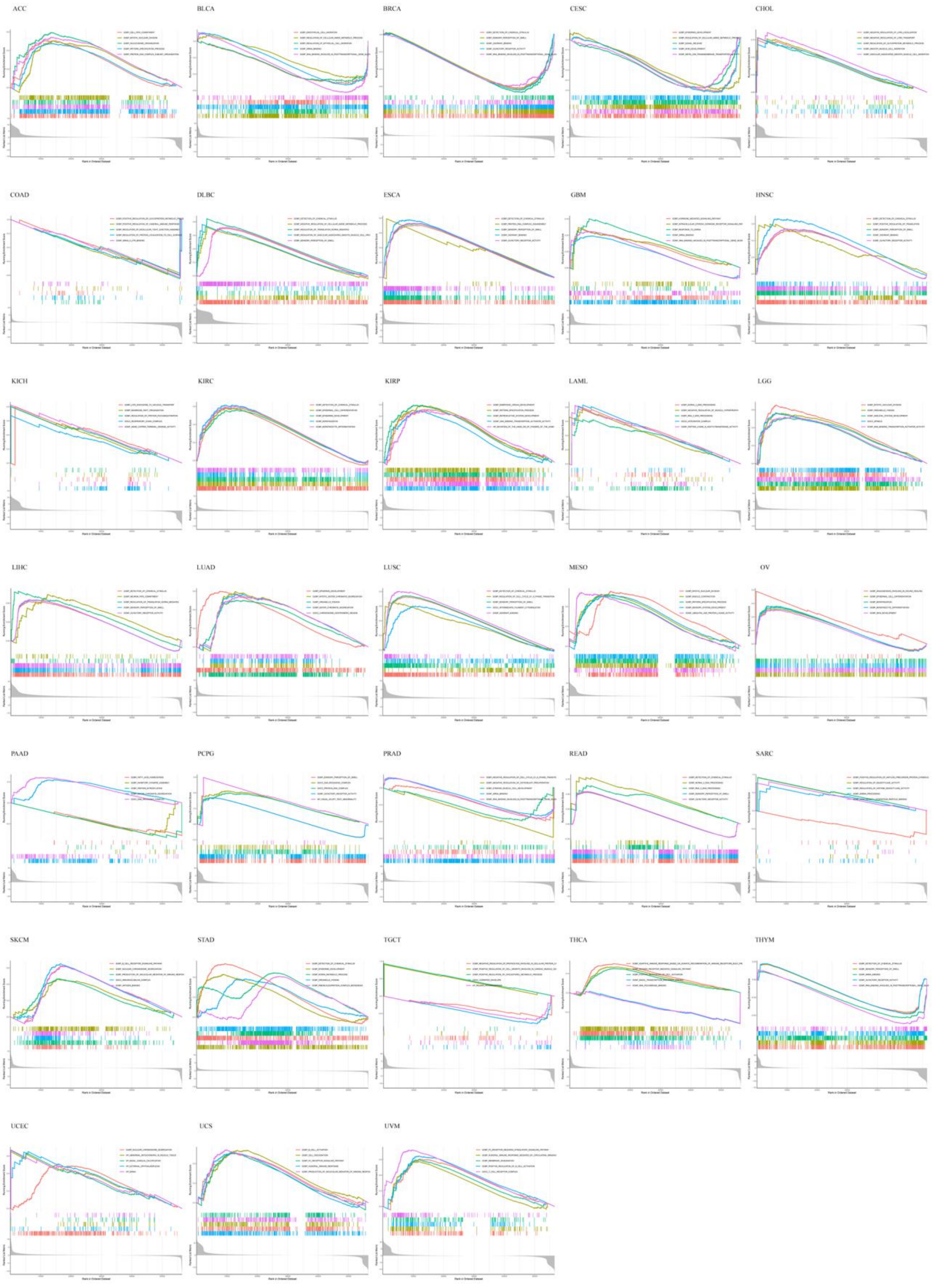

Supplementary Figure 5. GO enrichment plots from GSEA in various cancers. P< 0.05 and results higher than 5 were considered and shown.
