# Supplementary material for: RecQ mediated genome instability 2 (RMI2): a potential prognostic and immunological biomarker for pan-cancers
Source: Aging (Albany NY). 2022 May 12;14(9):4107–36. doi: 10.18632/aging.204076 (PMC9134953; doi:10.18632/aging.204076)
Supplement: Supplementary Tables 2-5 [file aging-14-204076-s003.pdf]

SUPPLEMENTARY TABLES

Supplementary Table 2. Correlation of *RMI2* expression in TIMER.

Tumor	Nor	pValue	sig
BLCA.Tumor	BLCA.Normal	3.33524769277125E-07	***
BRCA.Tumor	BRCA.Normal	3.51855503441419E-58	***
CHOL.Tumor	CHOL.Normal	2.2569207869384E-09	***
COAD.Tumor	COAD.Normal	5.09324209086511E-10	***
ESCA.Tumor	ESCA.Normal	1.47875599886395E-07	***
HNSC-HPVpos.Tumor	HNSC-HPVneg.Tumor	3.02521811314318E-24	***
HNSC.Tumor	HNSC.Normal	3.55755043752562E-20	***
KICH.Tumor	KICH.Normal	0.00126847525903987	**
KIRC.Tumor	KIRC.Normal	0.746778798029572	***
KIRP.Tumor	KIRP.Normal	0.0000642300913440225	***
LIHC.Tumor	LIHC.Normal	2.35042317884794E-19	***
LUAD.Tumor	LUAD.Normal	3.53592884726335E-33	***
LUSC.Tumor	LUSC.Normal	1.92132801669773E-31	***
PRAD.Tumor	PRAD.Normal	0.90674176451126	***
READ.Tumor	READ.Normal	0.000900238226685772	***
SKCM.Tumor	SKCM.Metastasis	0.0235685767763094	*
STAD.Tumor	STAD.Normal	8.16436195648269E-16	***
THCA.Tumor	THCA.Normal	9.64507732347014E-20	***
UCEC.Tumor	UCEC.Normal	7.41966534248929E-17	***

Supplementary Table 3. Results of correlation analysis and P values for the association between *RMI2* and tumor mutation burden in various types of cancer.

CancerType	cor	pValue	sig
ACC	0.474015205259224	0.0000102095020665298	***
BLCA	0.266678404655109	4.51370678357016E-08	***
BRCA	0.307034278888121	1.04233562697838E-22	***
CESC	-0.123506561658841	0.0368396195915519	*
CHOL	-0.000257632463363041	0.998810164094373	
COAD	0.0326815920802906	0.516679172301661	
DLBC	0.231152204836415	0.168176171327348	
ESCA	-0.0929628862437465	0.242318328446714	
GBM	0.0653463259393641	0.430067761367925	
HNSC	0.1271398414015	0.00473699922375918	**
KICH	0.0684510847642091	0.587957414329208	
KIRC	-0.0246316584562543	0.654741273092012	
KIRP	0.000356367841481593	0.995280486762928	
LAML	0.00312455237984684	0.980610291433608	
LGG	0.371409551306634	7.82582305859602E-18	***
LIHC	0.0190161294499724	0.719535140228386	
LUAD	0.327924117031034	4.49082824122193E-14	***
LUSC	0.154634505735056	0.000608342201765788	***
MESO	0.359789867992628	0.00112714058966601	**
OV	0.115526648062491	0.0570515322426661	
PAAD	0.433876536089132	2.61594165255277E-08	***
PCPG	0.0461029177958374	0.542300570879155	
PRAD	0.257457219022785	9.77318507810713E-09	***
READ	0.0404896437239661	0.644830768057326	
SARC	0.161043278197531	0.0134458162394394	*
SKCM	0.153122561419223	0.000924392601885563	***
STAD	0.295077751360235	7.91313018553446E-09	***
TGCT	0.0788559498938455	0.345782152772025	
THCA	0.0319962870841893	0.48341662229611	
THYM	-0.601754457872108	7.20873346273979E-13	***
UCEC	0.0888893275226402	0.041761681172524	*
UCS	-0.115915283262917	0.394914261071945	
UVM	0.0518414009658814	0.647891586242472	

Supplementary Table 4. Results of correlation analysis and P values for the association between *RMI2* and microsatellite instability in various types of cancer.

CancerType	cor	pValue	sig
ACC	0.190216531374014	0.0931322546714989	
BLCA	0.144486073612387	0.00344584783278446	**
BRCA	0.0602074343936691	0.0532830129665119	
CESC	-0.157253955427272	0.00617186416152031	**
CHOL	0.138738738738739	0.418210643263417	
COAD	0.10798692889622	0.0256528424783219	*
DLBC	0.0972723574943735	0.510717604915052	
ESCA	0.149061453297136	0.0599399388300867	
GBM	0.254025047965466	0.00164748830962498	**
HNSC	0.0890240071905552	0.0475248898811593	*
KICH	0.0201287277912859	0.873550494805262	
KIRC	0.0727768202000211	0.18390790274595	
KIRP	0.0719169853687164	0.226152107861546	
LAML	-0.0968440741739157	0.432078390191153	
LGG	0.0346885863674217	0.435302685382034	
LIHC	0.130359960440855	0.0121988018588046	*
LUAD	0.0863044889301134	0.0511998204536363	
LUSC	0.0798489161321026	0.0765171083685421	
MESO	0.0833215420093114	0.456742796601622	
OV	0.00654795803077039	0.914395891543287	
PAAD	0.0566557711921846	0.456445419193762	
PCPG	0.00687408069590928	0.927439658620535	
PRAD	0.0671868946145736	0.135509988766736	
READ	0.0195190962168938	0.81135079318818	
SARC	0.0771106407307831	0.221606750972099	
SKCM	-0.022064656833176	0.633993887949297	
STAD	0.149820380360236	0.00368313245169416	**
TGCT	-0.0401238491545789	0.625903853468981	
THCA	0.0690560718207178	0.126486512959018	
THYM	-0.0267603113788236	0.77361586315177	
UCEC	0.139518839727519	0.00117669281174837	**
UCS	-0.093988174633656	0.490818745023117	
UVM	0.133157509672065	0.23899416650693	

Supplementary Table 5. Correlation of *RMI2* expression with stromalscore and immunescore of tumor microenvironment in various types of cancer.

CancerType	Gene	StromalScore	ImmuneScore
ACC	RMI2	0.0000390858836040747	0.0000988831872576334
BLCA	RMI2	0.0226058475104757	0.343291414108804
BRCA	RMI2	1.30618696914016E-22	0.158568640503641
CESC	RMI2	0.00805145155352228	0.654717820439346
CHOL	RMI2	0.997012069871571	0.752393158944291
COAD	RMI2	0.000187464378117756	0.186598288068983
DLBC	RMI2	0.864266367325916	0.254284082182472
ESCA	RMI2	0.00831601667500962	0.0510010346847696
GBM	RMI2	0.000621376160062087	0.000115307479851331
HNSC	RMI2	1.32422886823392E-10	0.834335469356639
KICH	RMI2	0.00458390464504713	0.00628582932463523
KIRC	RMI2	0.0884215630989439	0.000054173081658311
KIRP	RMI2	5.91016914043202E-06	0.0000840771471387407
LAML	RMI2	0.0535126707555291	0.0364696592934527
LGG	RMI2	0.0260563526256745	0.00876993687473695
LIHC	RMI2	0.000140249718826792	0.247605753279734
LUAD	RMI2	0.00903966330192381	0.474893311090555
LUSC	RMI2	0	6.3938627912076E-10
MESO	RMI2	0.961824524842495	0.572480717900196
OV	RMI2	0.0000618493821902081	0.217220542603398
PAAD	RMI2	0.0143781499827078	0.412085416209786
PCPG	RMI2	0.752023218549627	0.827355722175881
PRAD	RMI2	0.00128294775453137	9.90443019902769E-06
READ	RMI2	0.000465508008036331	0.00113116133531009
SARC	RMI2	2.04187627241984E-07	0.0601511782676821
SKCM	RMI2	0.650678797470389	0.00131806765435742
STAD	RMI2	4.79620250049032E-10	0.36650275902808
TGCT	RMI2	0.0110490309955332	0.402793167080098
THCA	RMI2	0.00125138169856009	7.77753865373765E-10
THYM	RMI2	0.000295097022690746	0.0002994392122266
UCEC	RMI2	6.41298157977979E-09	0.0000386507116199285
UCS	RMI2	0.528239541243031	0.607312904499613
UVM	RMI2	0.0212172807416378	0.259862077088759
