# Supplementary material for: RecQ mediated genome instability 2 (RMI2): a potential prognostic and immunological biomarker for pan-cancers
Source: Aging (Albany NY). 2022 May 12;14(9):4107–36. doi: 10.18632/aging.204076 (PMC9134953; doi:10.18632/aging.204076)
Supplement: Supplementary Table 8 [file aging-14-204076-s006.pdf]

Supplementary Table 8. Results of correlation analysis and P values for the association between *RMI2* and DNA methyltransferases-related genes.

**Correlation analysis**				
**CancerType**	**DNMT3L**	**DNMT3B**	**DNMT3A**	**DNMT1**
ACC	-'0.200360137884253	0.257962964981071	0.563726277122637	0.625003719611013
BLCA	0.0449711242905621	0.265259435878117	0.256052477271622	0.393349878220686
BRCA	0.071114624544912	0.421178672042221	0.224888986319261	0.334889405891321
CESC	0.08646962225981	-'0.0254404127652313	0.00745856559530254	0.113435929218695
CHOL	0.0923379970128044	0.44037704731701	0.00100972624426258	0.25089881569288
COAD	0.0632807834792187	0.126738101850277	-'0.0553918852497293	0.255650713920203
DLBC	0.157736820398358	-'0.218459343749995	-'0.231538745712161	0.0725198244662193
ESCA	0.102943238206266	0.314184992951274	0.330123553827303	0.400419251731121
GBM	0.0536267678171985	0.27993064729223	0.269306177891037	0.547870570062861
HNSC	0.246173633185529	-'0.0557531002277002	0.496903174657355	0.488241522160467
KICH	0	0.182005406624913	-'0.022854836449119	0.345698848588994
KIRC	0.0619340158449149	0.0903580705603833	0.0419854651258464	0.266910185094237
KIRP	-'0.153497637414578	0.319643564591859	0.0718810858950348	0.348454630188953
LAML	-'0.0561465181635485	0.0979927033870698	-'0.00201112355658134	0.431886640427158
LGG	0.0822597850158437	0.545460047720756	0.319418913061989	0.605625982678902
LIHC	-'0.0706741740603424	0.452012240396248	0.388188745710834	0.61113107089067
LUAD	0.00420804049822417	0.483442749368663	0.312358896512922	0.427111446000193
LUSC	0.0533604080185585	0.2628021603256	0.304128018166799	0.32353462275135
MESO	-'0.0160136644616164	0.323944857807078	0.281931014601778	0.488121698071459
OV	0.0950709075224625	0.255185885591459	0.11730437553452	0.291147990729008
PAAD	0	0.0765694068445306	-'0.0262337914558009	0.203603611949245
PCPG	0	0.174212020916852	0.0590751254756609	0.187637810350673
PRAD	0.0243836460220345	0.469587121329304	0.266337910687691	0.310544313521008
READ	-'0.0626701692129527	0.158134296208773	0.0756035436723812	0.228550393168756
SARC	0.0708270356693686	0.185837014219512	0.062878557071112	0.506628032822399
SKCM	0.0754866062823602	0.18934527654778	0.0325515006231845	0.32699739044805
STAD	0.146548514965192	0.3187668335866	0.105736936668231	0.476994306270815
TGCT	0.0851152659278019	0.207745288499105	0.147159154634777	0.219824519303687
THCA	-'0.0212902646631144	-'0.28478383873121	-'0.0018190836688661	0.301634138559775
THYM	0.0231028317365836	0.558273821517738	0.527876414260584	0.242848110672563
UCEC	0.129723643504282	0.359400991960914	0.316612889877476	0.311703480284695
UCS	0.196709818676955	0.146097142180212	0.237317377303806	0.148054294222397
UVM	0.00653117210511927	0.100760723222183	0.0419253981940825	0.156031709555029
**P Value**				
**CancerType**	**DNMT3L**	**DNMT3B**	**DNMT3A**	**DNMT1**
ACC	0.0766514145359805	0.0217174090098931	6.324417746548E-08	7.43248935542309E-10
BLCA	0.363144769549215	4.77841951516398E-08	1.41591100574209E-07	1.16581141235908E-16
BRCA	0.0181176210250711	1.06153842894506E-48	4.00404421214799E-14	2.44152741576326E-30
CESC	0.131237935693897	0.657566197035078	0.896614338543596	0.0474120241005856
CHOL	0.592219847705629	0.00718989241132774	0.995336758352739	0.139946205194513
COAD	0.170350455654451	0.0058809910695463	0.230189305751099	1.83046325878134E-08

DLBC	0.284283346454967	0.135772970819782	0.113315532060386	0.624251426236082
ESCA	0.192379021977592	0.0000467025076140987	0.000017855359281409	1.29444093634322E-07
GBM	0.489949072192117	0.000237872122631501	0.000415572778868932	1.51881970936045E-14
HNSC	2.29592551516617E-08	0.212388644437826	1.16061401234138E-32	1.97468303902564E-31
KICH	1	0.14676500553476	0.856595310778017	0.00479398790561718
KIRC	0.152556106382204	0.0366739432847534	0.332404190589159	3.53308395288018E-10
KIRP	0.00895787042221523	2.74687312378113E-08	0.223125556966811	1.12922215090402E-09
LAML	0.493503522878231	0.231294291262527	0.98044759258048	3.07676320861546E-08
LGG	0.0586637568747953	2.44969784964295E-42	5.178572227458E-14	2.92650922306304E-54
LIHC	0.172597676809913	3.12368244195714E-20	6.71877569867905E-15	1.14304010271889E-39
LUAD	0.923297456512925	3.67316062014015E-32	2.28386601035014E-13	9.77791193495706E-25
LUSC	0.233169619492754	2.33043368723643E-09	3.50718980580238E-12	1.13463946340975E-13
MESO	0.883652278171976	0.00234482893937052	0.00854252686875455	1.86597377940176E-06
OV	0.064470917511177	4.77517368265002E-07	0.0223703652182252	7.69972407617676E-09
PAAD	1	0.309696859644671	0.728143302959535	0.00641291382440643
PCPG	1	0.0183433612313819	0.426979116359322	0.0109730038325147
PRAD	0.586851559209378	9.85005120860367E-29	1.50220069615795E-09	1.2858647475278E-12
READ	0.421058950583828	0.0412451402198517	0.331515689816005	0.00297062940444784
SARC	0.252379741264963	0.00247963584923971	0.309692621134178	1.48906399515356E-18
SKCM	0.10179088259885	0.0000353682831112207	0.480959802471325	3.36626741062782E-13
STAD	0.00445797990077616	2.65229041581928E-10	0.0407080541277055	1.05689301481445E-22
TGCT	0.290759072129588	0.00925941616639852	0.066768557966094	0.00582743319263733
THCA	0.631457457904058	5.6889154317383E-11	0.96731189966678	3.45506657373542E-12
THYM	0.803055057061937	4.20212466610946E-11	6.86778909902957E-10	0.00778723280648351
UCEC	0.00234477977576471	3.75246564189301E-18	3.17589299620528E-14	8.20722592675448E-14
UCS	0.146201414674964	0.282638947970259	0.0782180118886614	0.276170232077738
UVM	0.954148668402514	0.373834660125751	0.711939329981825	0.166938867151943
