# Supplementary material for: RecQ mediated genome instability 2 (RMI2): a potential prognostic and immunological biomarker for pan-cancers
Source: Aging (Albany NY). 2022 May 12;14(9):4107–36. doi: 10.18632/aging.204076 (PMC9134953; doi:10.18632/aging.204076)
Supplement: Supplementary Table 10 [file aging-14-204076-s008.pdf]

Supplementary Table 10. Results of correlation analysis and P values for the association between *RMI2* and MMR-related genes.

Correlation analysis					
**CancerType**	**EPCAM**	**PMS2**	**MSH6**	**MSH2**	**MLH1**
ACC	0.0821440537042775	0.0799740759949614	0.647836497426721	0.628063871055481	0.344504291698851
BLCA	0.343332657765153	0.131589028770143	0.418388772805255	0.474025984549608	0.187314564555575
BRCA	0.318305837118849	-'0.0983879215868585	0.398127137544394	0.358303635052018	-'0.0159458176642903
CESC	0.105503527792963	-'0.146097740510574	0.314837032089535	0.157176716517584	0.16310195242743
CHOL	0.0233895291459036	-'0.0917379016554158	0.00301074244521845	0.141954438034597	0.0265452288705128
COAD	0.232485128201342	0.0748879764361371	0.296105759372467	0.37125649239809	0.0756095107016791
DLBC	-'0.0739551656307502	-'0.143271248145248	0.415422717797911	0.189946427346192	0.0840220907555631
ESCA	0.0747728694554444	0.331637602236161	0.553564291847022	0.531205142587756	0.247902877854317
GBM	0.00945807531275092	0.251639296001945	0.581475285943948	0.619040926553936	0.381721368304047
HNSC	0.487813285892269	0.0820170208142909	0.661054936079977	0.592805727271638	0.409435886960755
KICH	0.0280372630540483	0.273393276179095	0.0765800030650024	0.228696749925432	0.432501582896325
KIRC	0.0595197523695539	0.094187692072875	0.178606902098665	0.119432549698141	0.219020106602401
KIRP	0.247839499612641	0.00123463653688213	0.401589231448369	0.388727144402393	0.226440355929117
LAML	0.00524966292184514	0.183289184323286	0.392707603800386	0.424600197758876	0.535266946961695
LGG	-'0.000596279130696958	0.171586119548268	0.598125160174282	0.606696301255872	0.546004543510865
LIHC	0.114828804603286	0.14585240789578	0.596743033176181	0.590240490002869	0.355090029283642
LUAD	0.092472631440778	0.187618636858392	0.488163293804794	0.491065504329307	-'0.0398878303829284
LUSC	0.343060701785532	0.198644270166663	0.443496675468319	0.480546175774448	0.0806476103773541
MESO	-'0.00499751121184967	-'0.0763933486525221	0.274547208618512	0.311347995408141	0.133817562239604
OV	0.135960188989067	-'0.0121401345221567	0.407755684576346	0.308477460222789	0.195483191496278
PAAD	0.153226348922255	0.0605848896532685	0.251832107683497	0.377481623102397	0.0617734273484252
PCPG	0.119780924551556	0.117369979427585	0.267967104135493	0.26294234629	-'0.000237584028312146
PRAD	0.122817719889412	0.0246177458537532	0.197201515715449	0.216236494588665	0.0876088536528323
READ	0.255877287220866	0.179480723855562	0.36201090085634	0.382880939113039	0.328499476408673
SARC	-'0.0151629017182021	-'0.0312884017204114	0.383802651522951	0.428342489073957	0.37877306529507
SKCM	0.0424725668527406	-'0.0132562599707591	0.276647636651524	0.2101548708644	0.22142881187743
STAD	0.262160626092101	0.194698166052425	0.316266110887768	0.449101368289907	-'0.0177656890963422
TGCT	0.249999156688096	-'0.0584971326370555	0.201848049356406	0.1895187252333	0.113745895248147
THCA	0.226952047433801	-'0.303235545180183	0.12072989815134	0.167925473146033	-'0.210306298554086
THYM	-'0.393925807919425	-'0.429515723887069	0.562085740652993	0.33985189492272	0.292924930222935
UCEC	0.103289458194565	0.149879083268749	0.532535898251585	0.471740811005193	0.078421623433428
UCS	0.245139079605699	0.00565875392056001	0.365552344325895	0.245004259436857	0.151130058568515
UVM	0.0903379453262149	0.0907859805375449	0.217953653235259	0.168797678189301	0.168249910514304
**P Value**					
**CancerType**	**EPCAM**	**PMS2**	**MSH6**	**MSH2**	**MLH1**
ACC	0.471714350782446	0.4835411069326	1.09488339682453E-10	5.80216577334858E-10	0.00187736275765539
BLCA	8.18839102300496E-13	0.00755756305466816	7.56706159819222E-19	2.05558857037946E-24	0.00013358709055725
BRCA	2.0476354314611E-27	0.00106289368556395	3.03390197603572E-43	8.78492591163656E-35	0.596627122893919
CESC	0.0653068325984739	0.0104988274386812	1.81635237768732E-08	0.00586269790270323	0.00422811108310687
CHOL	0.892293418033666	0.594638612249745	0.986096016360898	0.40888234432142	0.877864170666817
COAD	3.35756092843801E-07	0.104542326727945	5.49831924889005E-11	7.71316908775837E-17	0.101233159096261

DLBC	0.61738821842825	0.331313693681505	0.00332227968447292	0.195973774481482	0.570182257421975
ESCA	0.344322909875124	0.0000162507468452418	2.17377293777691E-14	3.53722612302815E-13	0.00146942739385958
GBM	0.903153945576466	0.00100052939518828	1.40219993651319E-16	3.77351604640458E-19	3.30100410121562E-07
HNSC	2.26694955907257E-31	0.0663378395457325	2.28675810027222E-64	5.87300769502783E-49	1.03264683062522E-21
KICH	0.82454648833846	0.0275549254637183	0.544302665873787	0.0668913055933976	0.000320667412950062
KIRC	0.169226094024357	0.0293824822443892	0.0000325312789971185	0.00567610967302796	3.11506111755969E-07
KIRP	0.0000202889933402856	0.983327129157925	1.2613536879562E-12	7.33117923372204E-12	0.000103021513803574
LAML	0.948991473145845	0.0242781844487876	6.11916489559317E-07	5.52439847360448E-08	1.43390828562046E-12
LGG	0.989083700415348	0.0000729050045302756	1.2358002540572E-52	1.70157039723921E-54	1.95807522229117E-42
LIHC	0.0263780728854646	0.00470835041998098	1.89871340714166E-37	1.76070269472917E-36	1.48332941659811E-12
LUAD	0.0339781813265242	0.0000148258990749927	7.54727284352726E-33	2.81788545660415E-33	0.361241473564211
LUSC	2.77612486934635E-15	7.46868087682243E-06	1.48168733275921E-25	2.6141956906616E-30	0.0712991349059968
MESO	0.963575571037041	0.484490357696298	0.0105231027013739	0.00352318805099024	0.219316691876976
OV	0.00803876626885212	0.8137654265647	1.29219154871943E-16	8.47037100214993E-10	0.000128093300116431
PAAD	0.0411548886148053	0.421774295927245	0.00069636112848981	2.05585601175305E-07	0.41270850602602
PCPG	0.106292620548349	0.113570569968096	0.000244976817420351	0.000322981387191133	0.997453193157385
PRAD	0.00601309363444804	0.583264340038719	9.09032916060072E-06	1.08231575548577E-06	0.0504783990308078
READ	0.000845153518016335	0.0202943968042674	1.53108245807383E-06	3.27827267207538E-07	0.0000146317238586325
SARC	0.806654873655104	0.613475007962911	1.1711825223003E-10	3.68305685571003E-13	2.13028638955916E-10
SKCM	0.357714848259828	0.774156276904201	1.01138274356665E-09	4.21992014703133E-06	0.0000012150325490843
STAD	2.60316618389226E-07	0.000148181894796472	3.71471525730289E-10	5.17334619473483E-20	0.731666452651868
TGCT	0.00164667397806354	0.468222416080093	0.0115084072195494	0.0178089389382712	0.157405109770611
THCA	2.21324598093013E-07	2.62203132502527E-12	0.00633792492089639	0.000138924875634883	1.65605351903604E-06
THYM	9.31680436847047E-06	1.09112470138272E-06	2.90092318446333E-11	0.000155944759097587	0.00122529367551104
UCEC	0.0155676969099672	0.000430778291146385	1.83316725747897E-41	1.02781915980838E-31	0.0665891277470141
UCS	0.0686109976117325	0.966983827188279	0.00559814102354201	0.0687682087408199	0.266204068377308
UVM	0.425496535478075	0.42319509909396	0.0521161350634573	0.134452163260153	0.135739561718928
